# South-Tyrolean pinot blanc identity: Exploration of chemical and sensory profile changes ascribed to vineyard locations and winemaking variables

**DOI:** 10.1016/j.fochx.2024.101824

**Published:** 2024-09-10

**Authors:** Aakriti Darnal, Simone Poggesi, Vakarė Merkytė, Edoardo Longo, Emanuele Boselli

**Affiliations:** a Oenolab, Free University of Bozen-Bolzano, Faculty of Agricultural, Environmental, and Food Sciences, Piazza Università 5, 39100 Bolzano, Italy; b Food Experience and Sensory Testing (Feast) Lab, Massey University, Palmerston North 4410, New Zealand; c Competence Center on Food Fermentations, Free University of Bozen-Bolzano

**Keywords:** Pinot blanc, Vineyard locations, Pre-fermentative grape freezing, Co-inoculation, Descriptive sensory analysis, Volatile and phenolic profile

## Abstract

The sensory and chemical properties of ‘Pinot Blanc’ wine from South Tyrol were studied in relation to vineyard location and winemaking technique. Musts and wines were collected from a local producer. Wines made with the same control vinification but from different vineyards (Aldino 800, Montagna 450, and Klaus 550 m.a.s.l) were analyzed. Then wines from grapes of the same vineyard but made with different vinifications (grape freezing and co-inoculation of yeast with malolactic bacteria, both compared against controls) were compared. The highest-altitude vineyard (cooler temperatures, increased UV radiation, and increased airflow) impacted positively the wine quality. The different vinifications produced differences at various storage times. Finally, sensory attributes predictors for the overall quality and related chemical variables were identified. As climate change pushes growers to increasingly high-altitude viticulture, if/when allowed by the environmental conditions, these results can contribute to understand which winemaking techniques are best in these more challenging conditions.

## Highlights

• Investigation of the typicity of Pinot Blanc wine from South Tyrol (Italy).
• Significant difference in sensory and chemical parameters due to vineyard location.
• Overall quality significantly higher in wines from Aldino vineyard.
• Chemosensory changes due to effect of grape freezing and co-inoculum.
• Important sensory and chemical variables predictors obtained for the wine quality.

## 1 Introduction

‘Pinot Blanc’, also known as ‘Weissburgunder’ or ‘Pinot Bianco’ in South Tyrol (Italy), originated from a mutation of ‘Pinot Noir’, the original variety of Burgundy grapes. Until the end of the 19th century, the vineyards were planted with a mixture of the ‘Chardonnay’ variety and the Pinot Blanc variety as they were not perceived as separate. Probably, in South Tyrol, the first planting of ‘Pinot Blanc’ took place in 1852 in Bressanone (https://www.altoadigewines.com/en/wine-varieties/pinot-blanc/22-12228.html, accessed on January 29, 2024). In recent years, ‘Pinot Blanc’ has become the fourth most planted variety in the region, covering about 588 ha of area out of 5657 ha of the region's vineyard surfaces (Statistiche | Chamber of Commerce Bolzano (camcom.bz.it), accessed on January 29, 2024). Apart from South Tyrol, ‘Pinot Blanc’ is also found in other regions of Italy such as Veneto and Lombardy. However, South Tyrol is considered one of the most important winemaking areas in Italy for Pinot Blanc.

‘Pinot Blanc’ variety is considered a neutral variety (non-aromatic). It is characterized by “yellow to gold”, sometimes even with a “greenish shimmer” colour. It has low acidity and has light to moderate body. “Apple”, “pear”, “yellow fruits”, “spicy” and “mango” are the sensory terms that have been used to describe its characteristic flavour (Rapp, 1998). In South Tyrolean ‘Pinot Blanc’ wines, typical aromas of “apple”, “pear”, “citrus”, and “green” notes; occasional “quince” and “exotic fruit” or “spicy” and “nutty” notes (due to storage in barriques) are very characteristic. Vineyards, where the soils are easily heated, are characterized by their so-called “typical” aromas and locations with lower annual average temperatures have fewer hints of apple and pear aromas (Pedri & Pertoll, 2013).

The wine character of ‘Pinot Blanc’ varies by its growing area. *Terroir* is an essential concept when it comes to final wine quality as it depends on the origin of the vine, the soil, the oenological practices and time of harvest (Michelini et al., 2021). *Terroir* is the all-encompassing ecosystem accounting for soil, climate, topography and vine along with the cultural practices that influence these variables and how the wine is produced (Van Leeuwen, 2015). This gives the wine its sensory uniqueness. The commercial value of the wine increases if the quality of the wine from a *terroir* is high which also becomes a very important economic tool in sales of the wine (Jackson, 2020). The concept of *terroir* is strong in South Tyrol, as 98 % of the produced wines are labelled as DOP (Protected Designation of Origin) or PGI (Protected Geographical Indication) (https://www.suedtirolerland.it/en/leisure-activities/food-and-drinks/south-tyrolean-wine/, accessed on May 14, 2024).

The growth of ‘Pinot Blanc’ is well suited to the alpine viticultural conditions of the mountain areas in South Tyrol. This variety is less susceptible to early frosts at higher altitudes and ripens later. Cool temperatures and higher fluctuations during the ripening period keep a better ratio of the grape ingredients (e.g. lower sugar levels and higher acidity) (Tscholl et al., 2019). South Tyrol has a wide elevation range with complex topography and many niches that are theoretically available for viticulture. The Huglin Index (HI) of South Tyrol (1700 < HI ≤ 2200) is reported to be similar to the range calculated for the cultivar itself (Balotti et al., 2018). According to Failla, 2008, South Tyrol has the best possibilities of viticulture expansion to higher altitudes. More than 50 % of new vineyards have been established above 500 m.a.s.l. and currently only 2 % of the wine-growing areas lay over 800 m.a.s.l. Generally, higher values of radiation intensity are observed in higher-altitude vineyards. The 2700 number of vineyards reportedly have an altitude of 498 ± 140 m.a.s.l (Ferretti, 2020; Tscholl et al., 2019).

The medium annual temperature in South Tyrol has increased by about 1.9 °C from 1920 to 2019 resulting in the expansion of areas suitable for viticulture at higher altitudes and the range of grape varieties and wine characteristics change in the existing cultivation areas (Tscholl et al., 2019).

This study thus investigated the identity of South Tyrolean ‘Pinot Blanc’ wines in relation to vineyard location and enological practice. As mentioned, differences in growing conditions and vinification methods typical for a particular region can lead to the creation of wines distinct from other growing areas. The chemical profile and the sensory perception of the samples and their oenological parameters were therefore examined. Three control vinifications for different vineyard locations have been studied. Additionally, two different experimental vinification theses (simultaneous malolactic and alcoholic fermentation and the effect of pre-fermentative grape freezing) have been considered for the same vineyard location.

## 2 Materials and methods

### 2.1 Vineyards, vinifications, and sampling

**Fig. 1:**
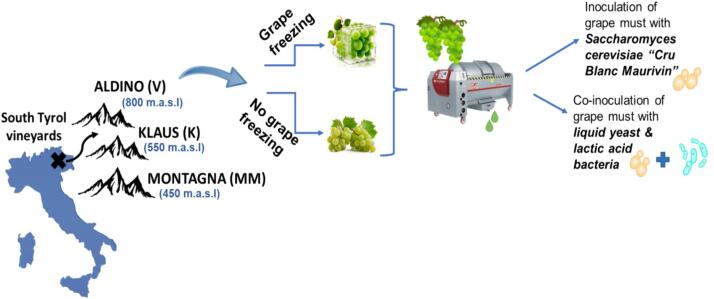
Scheme of the vineyards and vinification of ‘Pinot Blanc’ samples from South Tyrol.

The must and wine samples were sampled at Franz Haas Winery in Montagna (Italy), a small village in the Province of Bolzano. The ‘Pinot Blanc’ grapes were harvested from three different vineyards (Aldino, Klaus, and Montagna) located relatively near to the winery, but at different altitudes, which is represented in the schematic diagram below (Fig. 1**)**.

Musts (soft pressed; obtained with an applied pressure of up to max. 0.5 bar), and young wines (aged for 1 month after the completion of fermentation) were all sampled in triplicates. The samples were stored in 50-mL falcon tubes. After sampling, the tubes were immediately refrigerated, transported to the laboratory with the help of cool bags, and then readily analyzed. The musts sampling started on September 10th, 2020 for Montagna (MM), September 17th, 2020 for Klaus (K), and October 2nd, 2020 for Aldino (V) vineyards. For young wines, the samples from all different vinifications were sampled from November 4th, 2020 onwards. Additionally, bottled wine samples from different vineyards were provided and analyzed at different storage months (0, 6, and 12).

The crushed grapes were pressed before inoculation with yeasts. The must was separated at different pressure levels and treated differently but were put together at inoculation. The fermentation was carried out in stainless steel tanks at 16–18 °C in two different ways; inoculation with selected yeast or by co-inoculation of liquid yeast and lactic acid bacteria. For control variants, the vinification was similar, while the aging was different. The detailed winemaking process is reported in **Table S1**.

## 3 Analytical and statistical methods

### 3.1 Basic oenological parameters

MIURA One (Exacta&Optech, San Prospero, Modena, Italy), a multi-parametric wine analyzer was used for the evaluation of the basic oenological parameters such as glucose-fructose (g^.^L^−1^), tartaric acid (g^.^L^−1^), lactic acid (g^.^L^−1^), malic acid (g^.^L^−1^), acetic acid (g^.^L^−1^), ammonia nitrogen (mg^.^L^−1^), α-amino acids nitrogen (mg^.^L^−1^), free sulfur dioxide (mg^.^L^−1^), total sulfur dioxide (mg^.^L^−1^), and total polyphenols (mg^.^L^−1^) in samples of ‘Pinot Blanc’ (must or wine). Calibration curves against reference concentration standards were recorded. All the samples were filtered with a 0.22 μm syringe filter before the analysis (Tchouakeu Betnga et al., 2024).

### 3.2 Profiling of volatile compounds by HS-SPME-GCxGC-ToF/MS

The volatile compounds of the ‘Pinot Blanc’ samples were characterized using adapted methods from already published studies (Darnal et al., 2024; Dupas de Matos et al., 2020). For SPME sample preparation, each sample was made by adding 0.5 g of NaCl into a 10-mL SPME glass vial sealed with a perforable screwcap with a perforable silicon septum. Then, 4 mL of the sample was added into the vial, after which 10 μL of internal standard (IS) – i.e., a pre-diluted 2-methyl-3-pentanol solution (stock: 1:50 in ethanol) - was added. The samples were stored in a cooled rack (5 °C) endowed on the PAL-3 autosampler. For analysis, each sample was incubated at 40 °C for 15 min under stirring at 300 rpm. Then, a pre-conditioned triphasic SPME fiber (50/30 μm DVB/CAR/PDMS, Stableflex, 23 Ga, 1 cm – Sigma-Aldrich) was inserted in the sample headspace and kept under 300 rpm stirring (5 s on time, 2 s off time) at 40 °C for 15 mins. Finally, the sample was injected for analysis via the 240 °C heated GC split/splitless inlet.

Each sample was analyzed on a comprehensive GCxGC instrument coupled with a Pegasus BT 4D time-of-flight mass spectrometer, and equipped with Flux™ flow modulator (Leco Italy, Milano). The used carrier gas was helium. The sample pre-conditioned fiber was exposed for 6 min at 240 °C in the split/splitless inlet of the GC. A 6 mL^.^min^−1^ carrier gas flow was applied as inlet purge (after 720 s from analysis start), and a 2 mL^.^min^−1^ flow for septum purge. The injection was done in splitless mode and the separation was carried out at a 1 mL^.^min^−1^ flow rate (constant flow) on a polar MEGA-Wax Spirit column (PEG-phase) 40 m/0.18 mm/0.30 μm (first dimension, MEGA s.r.l.) and a Rxi-17 Sil 0.10 μm × 0.10 mm x (0.7 + 0.31) mm (second dimension, Restek S.r.l., Cernusco sul Naviglio, Italy). The two columns were connected by the Flux™ modulator (LECO), with a 15.92 psi auxillary diverting helium flow (enabling to achieve the same carrier-gas flow in both first and second dimension, and to the mass detector). The Flux™ modulator is designed to ensure that the diverting flow rate is automatically set to yield an equal flow in first dimension, *sec*ond dimension, and to the mass detector. The second column is mounted in a thermally isolated secondary oven inside the larger primary oven. The secondary oven was kept constantly at +5 °C above the primary oven temperature. The applied temperature ramp (main oven) was: 40 °C for 6 min, then 40 °C to 180 °C at 3 °C min^−1^, then 180 °C to 240 °C at 10 °C min^−1^, and 240 °C for 1 min. The modulation period was set at 2.5 s and the injection time at 0.08 s. The transfer line temperature was 250 °C and the ion source temperature was 250 °C. The detection was done on the pre-tuned time-of-flight (ToF) Pegasus BT 4D detector. The following parameters were applied: solvent delay = 0 min, acquisition rate = 150 spectra^.^sec^−1^, acquisition mass range = *m/z* 35–530, and extraction frequency = 32 kHz.

The obtained 2D chromatograms were then processed by the ChromaTOF® software (LECO Corporation, Germany, ver2021). Then, Target Analyte Finding (TAF) for extracting the peak areas of the compounds used for the statistical analysis. The parameters used in the TAF method are reported in **Table S2.**

### 3.3 Profiling of phenolic compounds using HPLC-DAD

The profile of the phenolic compounds in the samples was characterized by HPLC analysis. The method was slightly adapted from Dupas de Matos et al., 2020. The mobile phase for this analysis was formed by solvent A (0.1 % formic acid in degassed milliQ water) and solvent B (0.1 % formic acid in acetonitrile - LC grade). The gradient method applied was: 0–2.5 min 1 % B, 2.5–50 min 1–25 % B, 50–51 min 25–99 % B, 51–55 min 99 % B, 55–56 min 99–1 % B, 56–60 min 1 % B. The HPLC flow rate was 0.7 mL^.^min^−1^. The separation was carried out on an ODS column (Eurosphere II, C18 stationary phase, 250 mm × 4.6 mm × 5 μm, Knauer, LabService Analytica, Anzola dell'Emilia Bologna, Italy) installed on a Nexera X2 UHPLC system (Shimadzu, Milano, Italy) equipped with a UV–Vis diode array detector (DAD, sampling rate 12.5 Hz, time constant = 0.320 s, scan range = 200–800 nm, 1.2 nm slit width). The HPLC peaks were reported as integrated areas vs. retention times (min) using the automatic integration tools provided in the LabSolutions software (Shimadzu). The peaks alignment was done manually, and compared to automatic alignment allowed by an *in house* developed alignment tool (Ianeselli et al., 2024). A series of standard compounds were also injected to obtain reference retention times and external calibrations.

### 3.4 Identification of the phenolic compounds using HPLC-MS

HPLC-QqQ/MS was performed offline (from HPLC-DAD). The analyses were carried out on a UHPLC-QqQ/MS instrument (Agilent LC/TQ 6465 system) equipped with a 1260 Infinity II UHPLC quaternary pumps system, a 1260 Infinity II WR PDA detector, in series with an AJS ESI QqQ mass spectrometer. Formic acid used for preparing the mobile phase and the solvents were LC-MS grade. Water used in the mobile phase was prepared *in house* as reported in paragraph 3.3. The chromatographic method and column used for the separations were the same as described above, in the paragraph 3.3. The MS analyses were run in full scan in negative ionization mode, with mass range *m/z* 100–750. Instrumental parameters: scan time = 500, step size = 0.1 amu, fragmentor potential = 135 V, cell acceleration = 5 V, N_2_ gas temperature = 350 °C, N_2_ gas flow = 11 L^.^ min^−1^, sheath gas = 40 psi, auxiliary gas = 15 psi, capillary voltage = −3500 V negative ionization, nozzle voltage = −500 V, nebulizer pressure = 60 psi, sheath gas temperature = 350C, sheath gas flow = 10 L^.^min^−1^, scan rate = 1000 Da^.^s^−1^, resolution Q1 (FWHM) = 0.7.

### 3.5 Sensory analysis

For the sensory analysis, eleven panelists (64 % female, 36 % male, aged 41 ± 12 years old) were recruited among the academic staff and students at the Free University of Bozen – Bolzano (Bolzano, Italy). The participants were selected based on their sensory sensitivities and their ability to discriminate the differences in the sensory properties among the samples. An informed consent form was signed by the participants who agreed to join the sensory analysis sessions after the full explanation of the aim of the project. No monetary reward was given. The study was considered low risk since for each session about 32 mL of alcohol was consumed which were under the tolerance threshold. Hence, no ethical consent was required. After this, the panel received specific training (ISO 8586:2012) on how to recognize and evaluate each sensory descriptor using intensity scales reported by Dupas de Matos et al., 2020. The training was divided into two sessions: qualitative and quantitative. The training sessions lasted 16 h in total. In the qualitative session, the sensory descriptors were generated by the panel. After the completion of the training sessions, the panel was provided with detailed instructions on the definition of the descriptors and how to conduct the sensory evaluation. The complete list of sensory descriptors is reported in **Table S3**. The panel performance was also checked to investigate the consistency of each panelist and between the panelists (data not shown). This helped in identifying the inconsistent assessors within the panel and therefore, his/her data was not included in the evaluation.

The bottled wines were then evaluated according to the Quantitative Descriptive Analysis (QDA®, Tragon Corporation, Arlington, TX, USA), which is described in the UNI 10957:2003 procedure. The QDA sessions were held at bottling (T0), six months after bottling (T6), and twelve months after bottling (T12). The wine bottles were opened just before the analysis. 30 mL of the wine samples per glass at around 16 °C were tasted following a random square Williams Latin order (in triplicates) by the panelists in ISO glasses codified with a 3-digit number. Panelists were also provided with low-salt crackers and water to cleanse their palate between the samples (Poggesi, Darnal, et al., 2022).

### 3.6 Statistical analysis

All the statistical analyses were performed with XLStat add-on for Excel (Lumivero, Denver, CO, USA). One-way and two-way ANOVA (analysis of variance) models with Tukey's HSD (honestly significant difference) *post-hoc* tests were performed on the datasets to highlight which were the most significant variables differentiating the samples.

To see the effect of the vineyard locations on the volatile and phenolic compounds of the samples, Principal Component Analysis (PCA) was carried out separately at different storage times, and radar plots of all the sensory variables were reported to show how they changed for each sample at different storage times separately. The same approach was followed to see the effect of pre-fermentative grape freezing and simultaneous alcoholic and malolactic fermentation on the volatile and phenolic compounds of the samples from Aldino vineyard using PCA and the changes in the sensory attributes using radar plots. Furthermore, Partial Least-Squares regression (PLS-R) was employed to have an understanding of 1) which variables affected the quality of the product; 2) which volatile compounds affected the sensory olfactory sensory attribute (important variable influencing the model for overall quality) 3) which non-volatile compounds affected the visual and gustatory sensory attributes (important variable influencing the model for overall quality) (Darnal et al., 2024; Poggesi, Darnal, et al., 2022). Additional wine samples from vineyards Enn and Ursch were used as validation sets for the regression models.

## 4 Results

The Results section is divided into three parts. The first part deals with the musts (soft-pressed) and resulting young wine samples (aged for 1 month) for which a specific study of the effect of the vineyard on the chemical parameters was carried out. This part also describes the effect of pre-fermentative grape freezing and simultaneous alcoholic and malolactic fermentation on the oenological and chemical parameters for must and young wine samples from the Aldino vineyard only. ANOVA (analysis of variance) tests are presented for the different variables in **Table S6-S17**.

The second part deals with the bottled ‘Pinot Blanc’ wines, which were studied at different storage times (0, 6, and 12). The second part is also divided into two subsections where 1) the effect of the vineyards on the bottled wines composition was studied, and 2) the effect of pre-fermentative grape freezing and simultaneous alcoholic and malolactic fermentation only on the samples from the Aldino vineyard were studied.

The third part deals with the overall quality (defined with sensory analysis) attributed to the products, including the application of regression models aiming at describing the “overall quality” in terms of the sensory descriptors. In this part, regression models were further applied to investigate which variables majorly influenced the different sensory attributes that have been identified as the most important in defining the wine profile.

The list of all volatile compounds determined in the musts and wines is reported in **Table S4.** An example of the two-dimension chromatogram which shows the identified volatile species in a must and a wine sample by comprehensive GCxGC-MS analysis is reported in **Figs. S1 and S2** along with some examples of the mass spectra of few identified compounds reported in **Figs. S3-S9**. Additionally, the list of tentatively identified phenolic compounds is reported in **Table S5** and the PDA spectrum and Full -MS spectra are reported in **Figs. S10 – S13.** Only the phenolic compounds that have been (at least) tentatively identified have been further discussed in this section.

### 4.1 Musts and young wines

#### 4.1.1 Effect of vineyard location on the oenological and chemical parameters applying control vinification conditions

The oenological parameters measured for musts before inoculation and young wines for all vineyards are reported in **Tables S6 & S7**. From the results, it can be observed that the concentration of sugars (glucose+fructose) was similar for the musts from vineyards Klaus and Montagna (**Table S6**), but for Aldino it was the highest. The tartaric acid content ranged between 1.92 g^.^L^−1^ for Aldino musts, 2.62 g^.^L^−1^ for Montagna musts, and 2.82 g^.^L^−1^ for Klaus musts. This showed that the amount of tartaric acid in the musts from Montagna and Klaus (the vineyards from similar altitudes) were in the same range and the highest. Malic acid values ranged between 3.07 g^.^L^−1^ to 3.79 g^.^L^−1^. The highest values were found in the must samples from Montagna and Klaus and were very similar, while the must samples from Aldino were comparatively lower.

For the young wines (**Table S7**) the residual sugar concentrations were below the detection threshold. The tartaric acid range was instead similar to that of the must samples. The content of acetic acid was also relatively low for all the samples, ranging from 0.18 g^.^L^−1^ to max. 0.31 g^.^L^−1^. The malic acid concentration was higher in samples from Aldino compared to the other samples. No lactic acid could be detected in these control samples (no inoculation of lactic acid bacteria). The total and free sulfur dioxide ranged from 53 mg^.^L^−1^ to 76 mg^.^L^−1^ and from 11 mg^.^L^−1^ to 13 mg^.^L^−1^ due to the addition of potassium metabisulfite during the vinification stage, and the activity of the yeast during fermentation. The total polyphenols ranged between 328 and 445 mg^.^L^−1^ but no clear correlation to the investigated variables was observed.

The results of a one-way ANOVA test applied to the datasets of volatile and phenolic compounds for musts and young wines are reported in **Tables S8-S11.** This ANOVA tests considered the differences between samples considering the vineyard location.

There was a significant difference in the volatile profile of the musts from different vineyards. Ethyl acetate (I), isoamyl alcohol (IX), 1-hexanol (XIII), 2-hexenol (XVII), 2,3-butanediol (XXIX), 2-phenylethyl acetate (XXXIX), benzyl alcohol (XLIV), and ethyl palmitate (LIV) were found to be significantly higher in the must samples from Montagna than the in other must samples while compounds, ethyl butanoate (II), hexanal (III), isoamyl acetate (V), 2-hexenal (VI), *cis*-3-hexenol (XV), benzaldehyde (XXVI), and β-damascenone (XL) were significantly higher in the samples from Aldino vineyard.

For the young wines, there were also significant differences in the volatile profiles across the different vineyards. Compounds isoamyl octanoate (XXXII), ethyl caprate (XXXIII), ethyl dodecylate (XLI), tetradecanol (LII), farnesyl acetate (LIII), and ethyl E-11-hexadecenoate (LV) were significantly higher in the samples from Klaus. Compounds ethyl 9-decenoate (XXXV), geranyl acetate (XXXVII), 2-phenylethyl acetate (XXXIX), β-damascenone (XL) were higher in the samples from Montagna. For Aldino, only styrene (X) was found to be significantly higher than in other samples.

The phenolic composition in the must samples were found to be the same for all vineyards. Only compounds x33 (*trans-*caftaric acid – *m/z* 311 at 28.2 min), x40 ((+)-catechin – *m/z* 289 at 34.0 min), and x52 (astilbin – *m/z* 449 at 50.0 min) were significantly different, with these compounds being higher in the must samples from Aldino vineyard.

However, in the case of young wines many significant phenolic compounds were found. In Klaus vineyard, compounds x34 (characterized by an in-source fragment *m/z* 129 eluting at 4.3 min), x47 (*m/z* 477 at 42.1 min), and x52 (astilbin) were significantly higher. Compounds x23 (*m/z* 218 at 22.4 min), x27 (protocatechuic acid, hexoside-*m/z* 315 at 24.2 min), x33 (*trans-*caftaric acid), x36 (*m/z* 175 at 31.5 min), x40 ((+)-catechin), x42 (*trans*-caffeic acid- *m/z* 179 at 36.6 min), and x48 (*m/z* 197 at 45.1 min) were found to be significantly higher in samples from Aldino. In full-scan MS mode, species x34 showed fragments *m/z* 129 and *m/z* 175, with their precursor ion being *m/z* 373; however, its definite assignment could not be provided (Poggesi, Darnal, et al., 2022). Ion x47 showed a precursor ion *m/z* 477 with a fragment ion of *m/z* 301, which was accordingly tentatively assigned to quercetin-3-*O*-glucuronide (Castillo-Muñoz et al., 2009). The species x23 at 22.4 min showed a likely precursor ion *m/z* 391 in negative mode with fragment ions *m/z* 241 with additional product ions *m/z* 169 and *m/z* 125 seen in the full MS spectrum and was speculated to be an analog of aucubin or a related species (Poggesi, Darnal, et al., 2022). Compound x27 (*m/z* 315 at 24.2 min) was tentatively identified as protocatechuic acid hexoside with the presence of fragment ion *m/z* 153 (Costa-Pérez et al., 2023), while compound x36 (*m/z* 175 at 31.5 min) was reported in previous studies as a fragment ion of *m/z* 373 but could not be identified as mentioned before; however, a possible hypothesis would be that this compound is a derivative of ascorbic acid or dehydroascorbic acid condensed with carbon monoxide (Darnal et al., 2024). The specie x48 showed precursor ion *m/z* 197 with product ion *m/z* 169 (due to loss of ethyl group) in the full MS spectra and was speculated to be ethyl gallate (Darnal et al., 2024). In samples from Montagna, compounds x19 (*m/z* 391 at 17.1 min), x20 (gallic acid- *m/z* 169 at 17.5 min), and x24 (*m/z* 143 at 22.6 min) were significantly higher than other samples.

#### 4.1.2 Effect of pre-fermentative grape freezing and simultaneous alcoholic and malolactic fermentation on the oenological and chemical parameters (for Aldino vineyard samples)

The oenological parameters measured for musts before inoculation for only samples from Aldino vineyard are reported in **Table S12**. In the case of Aldino vineyards, natural (control) musts were compared with those subjected to grape freezing. The samples with pre-fermentative grape freezing had higher sugar concentrations than the control sample because the pressing could have taken place before completely defrosting the sample. The amount of tartaric acid was higher in the control samples. Lactic acid could not be detected for any sample. Malic acid however was higher in the samples in which the grapes were frozen. Acetic acids were also very low and alfa-amino nitrogen was higher in the sample where the grapes were frozen while ammonia nitrogen was higher in the control sample.

For the young wines (**Table S13**), both pre-fermentative grape freezing and simultaneous alcoholic and malolactic fermentation were considered. The wines obtained with pre-fermentative grape freezing were found to have a relatively high amount of residual sugar compared to the non-frozen samples. Tartaric acid was comparatively higher in the non-frozen samples as well. The content of acetic acid was relatively low well within the accepted range. As expected, the malic acid content was lower for the samples with co-inoculation of lactic acid bacteria and the lactic acid was higher as the malic acid is converted to lactic acid during malolactic fermentation. The amount of both total and free sulfur dioxide was comparatively higher in the non-frozen samples. The total polyphenols were found to be in a similar range for all samples.

The ANOVA test results applied to the volatile compounds dataset for musts and young wines are reported in **Tables S14 & S15**. One-way ANOVA was carried out for the must samples to see the effect of grape freezing on the volatile compounds. The results showed that compounds 1-hexanol (XIII), 2-hexenol (XVII), 1-decanol (XXXVIII) and benzyl alcohol (XLIV) were significantly higher in the samples where the grapes were frozen and compounds ethyl butanoate (II), hexanal (III), isoamyl acetate (V), 2-hexenal (VI), and β-damascenone (XL) were higher in the samples where the grapes did not undergo freezing.

A two-way ANOVA was carried out for the young wine samples from Aldino vineyards to see both the effect of pre-fermentative grape freezing and the co-inoculation of liquid yeast and lactic acid bacteria on the volatile compounds. The volatile profile of the young wines was observed to be significantly different considering the interaction of the two factors. The volatile compounds ethyl butanoate (II), ethyl hexanoate (VII), hexyl acetate (XI), 1-hexanol (XIII), isoamyl octanoate (XXXII), ethyl succinate (XXXIV), and ethyl 9-decenoate (XXXV), were significantly higher in NF_LL samples. In samples NF_C, ethyl *trans*-hex-3-enoate (VIII) compound was significantly higher than in NF_LL samples. Ethyl octanoate (XIX) was higher in F_LL samples compared to NF_LL samples. And compounds such as 1-hexanol (XIII), 2-hexen-1-ol (XVII), and linalool (XXX) were higher in F_C samples.

The phenolic profiles in the musts from Aldino vineyard were analyzed by one-way ANOVA (**Table S16**). The investigated factor was the application for grape freezing in musts. There was no significant difference in most of the phenolic profiles of the must samples with grape freezing and no-grape freezing effect. However, the compounds x29 (S-glutathionyl caftaric acid (GRP))- *m/z* 616 at 24.8 min), x33 (*trans-*caftaric acid), x38 (*cis-*coutaric acid*-m/z* 295 at 33.3 min), x40 ((+)-catechin), x45 ((−)-epicatechin- *m/z* 289 at 38.5 min), and x47 (quercetin-3-*O*-glucuronide) were found to be higher in the samples from non-frozen grapes.

In the young wine samples, the phenolic profiles were analyzed by two-way ANOVA (**Table S17**), to see the effect of both pre-fermentative grape freezing and inoculation with *Saccharomyces cerevisiae* yeast or co-inoculation with liquid yeast and lactic acid bacteria. In these samples, many compounds were found to be significantly different. In samples NF_C, compounds x17 (*m/z* 317 at 12.8 min), x33 (*trans-*caftaric acid), x36 (*m/z* 175 at 31.5 min), x42 (*trans*-caffeic acid), x45 ((−)-epicatechin), and x47 (quercetin-3-*O*-glucuronide) were significantly higher compared to others. Compound x17 with precursor ion *m/z* 317 in full MS spectra showed the presence of fragment ions *m/z* 287 and *m/z* 151 and was tentatively assigned to myricetin (Abu-Reidah et al., 2015; Delgado De La Torre et al., 2015). In samples NF_LL, compound x49 (*m/z* 165 at 45.8 min) was significantly higher. In samples F_LL, the phenolic compounds x19 (*m/z* 391 at 17.1 min), and x27 (protocatechuic acid, hexoside-*m/z* 315 at 24.2 min) were higher.

### 4.2 Bottled wines

#### 4.2.1 Effect of vineyard location on the sensory and chemical parameters applying control vinification conditions

**Fig. 2:**
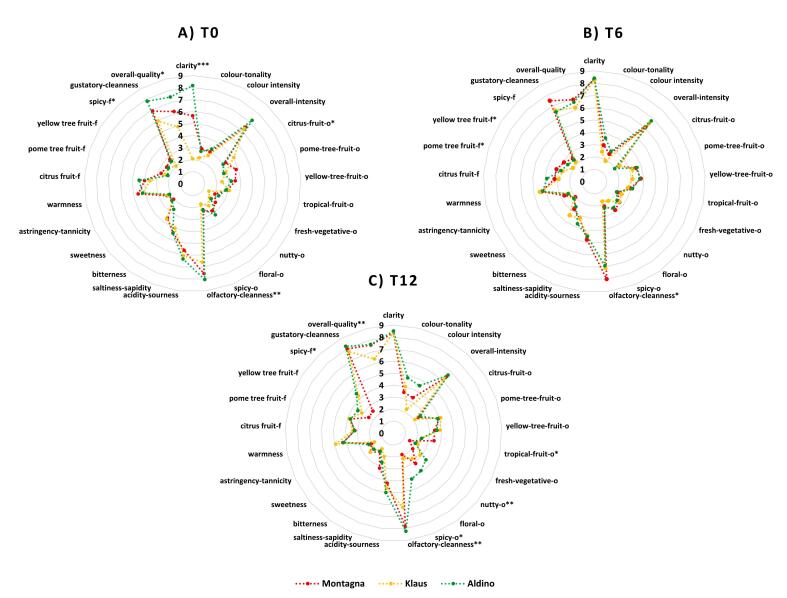
**(A-C).** Radar plots of the sensory attributes at different storage times (0, 6 and 12 months) for wines from different vineyard locations (*p < 0.05; **p < 0.01; ***p < 0.001).

The changes in the sensory attributes at storage time 0, 6, and 12 months for the wines from different vineyard locations are shown separately as radar plot in Fig. 2**(A-C)**. A one-way ANOVA performed on the sensory dataset of the samples from different vineyards is reported in **Table S18-S20.** At T0, the sensory attribute “citrus-fruit” aroma was significantly higher in the samples from Klaus vineyard while “spicy” flavour was higher in samples from Montagna vineyard. The attributes such as “clarity”, “olfactory-cleanness”, and “overall quality” were in turn significantly higher in wines from Aldino vineyard. At T6, again “olfactory cleanness” was significantly higher in the wines from Montagna vineyard along with “pome tree-fruit” flavour and “yellow tree-fruit” flavour. Finally at T12, “tropical-fruit” aroma was significantly higher in the samples from Montagna vineyard with attributes such as “nutty” aroma, “spicy” aroma, “olfactory cleanness”, “spicy” aroma and “overall quality” found to be significantly higher in the wines from Aldino vineyard.

**Fig. 3:**
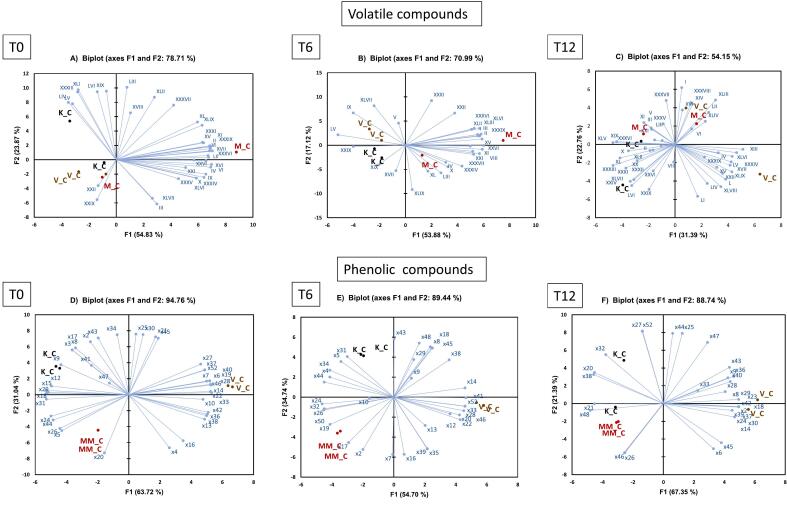
Principal Component Analysis biplots of the volatile datasets (**A-C**) and phenolic datasets (**D—F**) of wine samples from different vineyard locations at storage times 0, 6, and 12 respectively. M = Montagna; K=Klaus and V = Aldino.

PCA models were built for the ‘Pinot Blanc’ wine samples with the dataset of volatile compounds, and phenolic compounds to study the effect of vineyard locations. In this study, the PCA was conducted for datasets at each storage time; 0, 6, and 12 months (Fig. 3**)** separately to better visualize the effect of factors other than time evolution (Poggesi, Merkytė, et al., 2022). At T0, the volatile compounds that correlated mostly with the samples from Klaus vineyards were ethyl octanoate (XIX), ethyl caprate (XXXIII), ethyl dodecylate (XLI), ethyl palmitate (LIV), ethyl E-11-hexadecenoate (LV), and ethyl palmitoleate (LVI). For Montagna, the volatile compounds that characterized them were ethyl acetate (I), ethyl butanoate (II), isoamyl acetate (V), 2-hexenol (XVII), acetic acid (XXII), 2,3-butanediol (XXIX), n-octanol (XXXI), butenolide (XXXVI), and tetradecanol (LII). At T6, the samples from Klaus vineyard were mostly correlated to 2-hexenol (XVII), ethyl octanoate (XIX), and 2,3-butanediol (XXIX). The wine from Montagna was characterized by ethyl butanoate (II), hexanal (III), isobutanol (IV), ethyl *trans*-hex-3-enoate (VIII), styrene (X), hexyl acetate (XI), *cis*-3-hexenol (XV), benzaldehyde (XXVI), 3-octenol (XXI), ethyl succinate (XXXIV), geranyl acetate (XXXVII), 2-phenylethyl acetate (XXXIX), geraniol (XLII), isoamyl decanoate (XLIII), and phenylethyl alcohol (XLVI) while the compounds isoamyl acetate (V), isoamyl alcohol (IX), 2-phenylethyl butyrate (XLVII), and ethyl E-11-hexadecenoate (LV) characterized the wines from Aldino. While at T12, the volatile compounds that characterized the wines from Klaus vineyard were styrene (X), ethyl octanoate (XIX), isopentyl hexanoate (XX), ethyl hexanol (XXIV), benzaldehyde (XXVI), n-octanol (XXXI), ethyl caprate (XXXIII), butenolide (XXXVI), β-damascenone (XL), geraniol (XLII), 2-phenylethyl butyrate (XLVII), and ethyl palmitoleate (LVI). The compounds that characterized the wines from Montagna were hexanal (III), isoamyl acetate (V), 2-hexenal (VI), hexyl acetate (XI), ethyl 9 -decenoate (XXXV), geranyl acetate (XXXVII), isoamyl decanoate (XLIII), benzyl alcohol (XLIV), and tetradecanol (LII), while the compounds such as ethyl acetate (I), isobutanol (IV), 1-hexanol (XIII), *cis*-3-hexenol (XV), nonanal (XVI), 2-hexenol (XVII), acetic acid (XXII), ethyl succinate (XXXIV), phenylethyl alcohol (XLVI), ethyl palmitate (LIV), and ethyl E-11-hexadecenoate (LV) characterized the wines from Aldino.

In case of the phenolic compounds (Fig. 3D-F), at T0 the tentatively identified compounds x24 (*m/z* 143 at 22.6 min), x18 (gallic acid, hexoside - *m/z* 331 at 13.2 min), x17 (myricetin), and x34 (*m/z* 129 at 4.3 min) were positively correlated with the samples from Klaus, while Montagna was characterized by x20 (gallic acid). The samples from Aldino vineyards were mostly correlated with the phenolic compounds x19 (*m/z* 391 at 17.1 min), x33 (*trans*-caftaric acid), x27 (protocatechuic acid, hexoside- *m/z* 315 at 24.2 min), and x52 (astilbin). At T6, the phenolic compounds that characterized the wines from Klaus were x34 (*m/z* 129 at 4.3 min), x45 ((−)-epicatechin), and x48 (ethyl gallate). The compounds x17 (myricetin), x19 (*m/z* 391 at 17.1 min), and x38 (*cis-*coutaric acid) characterized the wines from Montagna while the compounds x20 (gallic acid), x33 (*trans-*caftaric acid), x41 (*m/z* 464.1 at 35.5 min) and x52 (astilbin) were more characteristic of samples from Aldino. Lastly, at T12, the phenolic compounds that characterized the samples from Klaus vineyard were, x38 (*cis-*coutaric acid), and x47 (quercetin-3-*O*-glucuronide). The samples from Montagna were instead characterized by phenolic compounds x21 (*m/z* 499 at 17.7 min) and x48 (ethyl gallate). The compounds that characterized the vineyard Aldino were x24 (*m/z* 143 at 22.6 min), x33 (*trans-*caftaric acid), x42 (*trans*-caffeic acid), x29 (GRP) and x40 ((+)-catechin).

#### 4.2.2 Effect of pre-fermentative grape-freezing and simultaneous alcoholic and malolactic fermentation (Aldino vineyard)

**Fig. 4:**
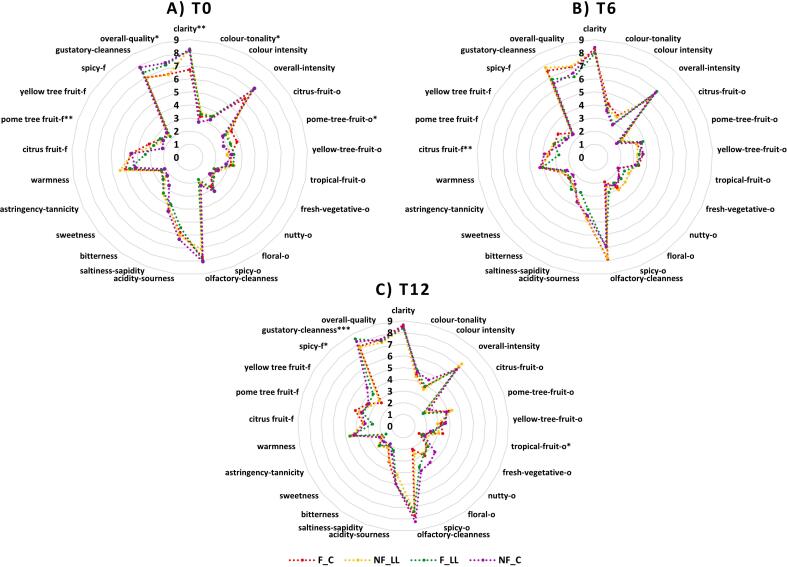
**(A-C)** Radar plots of the sensory attributes at different storage times for Aldino wines obtained via vinifications applying the different factors (*p < 0.05; **p < 0.01; ***p < 0.001). Legend: F: Frozen grapes, NF: Non-frozen grapes, C: Control/Alcoholic fermentation only, LL: Simultaneous alcoholic and malolactic fermentation.

The changes in the sensory characteristics at storage time 0, 6, and 12 of the wines from Aldino vineyard, which were treated with different factors are shown separately as radar plots in Fig. 4**(A-C)**. The two-way ANOVA tests performed on the sensory dataset of the samples from Aldino vineyard are reported in **Table S21-S23**. At T0 storage time, only sensory attributes such as “clarity”, “colour-tonality”, “pome-tree fruit” aroma and the overall quality were significantly different in the samples. The “clarity” attribute was significantly lower in the samples were the grapes were frozen and underwent alcoholic fermentation (F_C) compared to the rest of the samples while “colour-tonality” which represented colour from straw yellow to amber was significantly different for samples in which the grapes were not frozen and underwent alcoholic fermentation (NF_C) and was attributed as more straw yellow. Both the olfactory and gustatory attribute “pome-tree fruit”, were significantly different than each other. For the aroma, only F_C and NF_C samples were significantly different from each other and the frozen sample had more of this attribute than the non-frozen sample. The sample where the grapes were not frozen and underwent simultaneous alcoholic and malolactic fermentation (NF_LL) was significantly different than samples that underwent only alcoholic fermentation (NF_C) and also the F_C samples. The overall quality was also significantly different for the samples with the highest score given to the samples NF_C. At T6 storage time, only the “citrus fruit” flavour was significantly different in the F_C, F_LL and NF_C samples (highest score). Finally, at T12 storage time, only the attributes “tropical fruit” aroma, “spicy” flavour and “gustatory cleanness” were significantly different in the samples. Additionally, even though not significant for all time points, both olfactory and gustatory cleanness, overall intensity, clarity and the overall quality were always higher.

**Fig. 5:**
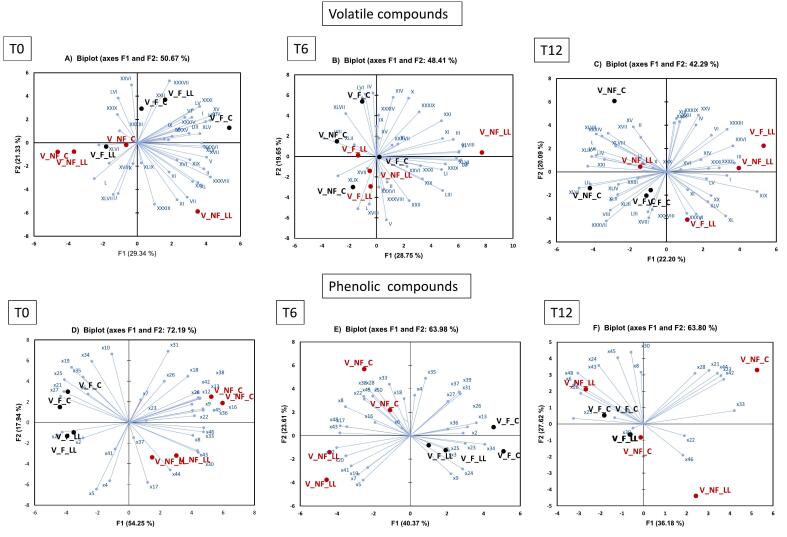
Principal Component Analysis biplots of the volatile datasets (**A-C**) and phenolic datasets (**D—F**) of wine samples from Aldino vineyard at storage times 0, 6 and 12 respectively. F: Frozen grapes, NF: Non-frozen grapes, C: Control/Alcoholic fermentation, LL: Simultaneous alcoholic and malolactic fermentation.

The effects of grape freezing (F vs NF), and co-inoculum (C vs LL) were then analyzed using PCA on the volatile profile of bottled wines from Aldino vineyards. Fig. 5 **(A-C)** shows the biplot at each storage time (0, 6, and 12). No clear effect of grape freezing nor the type of co-inoculum was observed in the volatile profile of these wines at all storage times. At storage month T0, some effect of grape freezing could be observed. The samples where grapes were frozen were characterized by the compounds ethyl acetate (I), 2-hexenal (VI), cis-3-hexenol (XV), acetic acid (XXII), benzaldehyde (XXVI), 2,3-butanediol (XXIX), n-octanol (XXXI), geranyl acetate (XXXVII), phenylethyl alcohol (XLVI), farnesyl acetate (LIII), ethyl palmitate (LIV), ethyl E-11-hexadecenoate (LV), and ethyl palmitoleate (LVI). The samples where the grapes were not frozen were instead characterized by the compounds ethyl butanoate (II), hexanal (III), ethyl hexanoate (VII), hexyl acetate (XI), nonanal (XVI), ethyl octanoate (XIX), 3-octenol (XXI), 1-decanol (XXXVIII), 2-phenylethyl acetate (XXXIX), and β-damascenone (XL). At T6, no clear trend of separation among the samples could be observed. At T12, however, again, some effect of the type of the co-inoculum was observed. The samples where only alcoholic fermentation was done were characterized by ethyl butanoate (II), ethyl trans-hex-3-enoate (VIII), cis-3-hexenol (XV), 2-hexenol (XVII), geranyl acetate (XXXVII), geraniol (XLII), benzyl alcohol (XLIV), 2-phenylethyl butyrate (XLVII), phenol (XLVIII), octanoic acid (XLIX), farnesyl acetate (LIII), and ethyl palmitoleate (LVI). The samples that underwent both alcoholic and malolactic fermentation were instead correlated with compounds such as ethyl acetate (I), hexanal (III), isoamyl acetate (V), 2-hexenal, (VI). hexyl acetate (XI), ethyl octanoate (XIX), isopentyl hexanoate (XX), benzaldehyde (XXVI), n-octanol (XXXI), isoamyl octanoate (XXXII), ethyl caprate (XXXIII), butenolide (XXXVI), β-damascenone (XL), and ethyl E-11-hexadecenoate (LV).

For the phenolic compounds, the effect of both pre-fermentative grape freezing and the use of co-inoculum (Fig. 5 **(D—F)**) are evident at T0 storage time. There was a clear separation considering PC1 and PC2, with replicates well-grouped together. The samples F_C were characterized by compounds x19 (*m/z* 391 at 17.1 min), x21 (*m/z* 499 at 17.7 min), x24 (*m/z* 143 at 22.6 min), x34 (*m/z* 129 at 4.3 min) and x35 (*m/z* 443 at 31.2 min) while the samples NF_C were more characteristic of x18 (gallic acid, hexoside), x36 (*m/z* 175 at 31.5 min), x38 (*cis-*coutaric acid), x42 (*trans*-caffeic acid), x45 ((−)-epicatechin) and x22 (*m/z* 161 at 20.7 min). Samples F_LL were characterized by phenolic compounds x24 (*m/z* 143 at 22.6 min), and x41 (*m/z* 464 at 35.5 min), while samples NF_LL were more characteristic of compounds x17 (myricetin), and x44 (*m/z* 131 at 37.5 min).

At storage time T6, the effect of pre-fermentative grape freezing was more evident. The samples in which the grapes were subjected to freezing were clustered together while the samples in which the samples were not frozen were grouped separately according to the type of fermentation (inoculum) applied. Both F_C and F_LL samples were characterized by phenolic compounds x24 (*m/z* 143 at 22.6 min), x35 (*m/z* 443 at 31.2 min), x23 (*m/z* 218 at 22.4 min), x34 (*m/z* 129 at 4.3 min), and x36 (*m/z* 175 at 31.5 min). The samples NF_LL were characterized by compounds such as x19 (*m/z* 391 at 17.1 min), x41 (*m/z* 464 at 35.5 min), and x20 (gallic acid). While the samples NF_C were more characteristic of compounds x22 (*m/z* 161 at 20.7 min), x38 (*cis-*coutaric acid), x45 ((−)-epicatechin), x33 (*trans*-caftaric acid), and x18 (gallic acid, hexoside). At T12 storage time, no specific trend of separation among the samples could be observed. The samples in which the grapes were not frozen were characterized by compounds x48 (ethyl gallate), x24 (*m/z* 143 at 22.6 min), x45 ((−)-epicatechin), x21 (*m/z* 499 at 17.7 min), x44 (*m/z* 131 at 37.5 min), x23 (*m/z* 218 at 22.4 min), x42 (*trans*-caffeic acid), x33 (*trans*-caftaric acid), and x22 (*m/z* 161 at 20.7 min).

### 4.3 Overall quality judgement

**Fig. 6:**
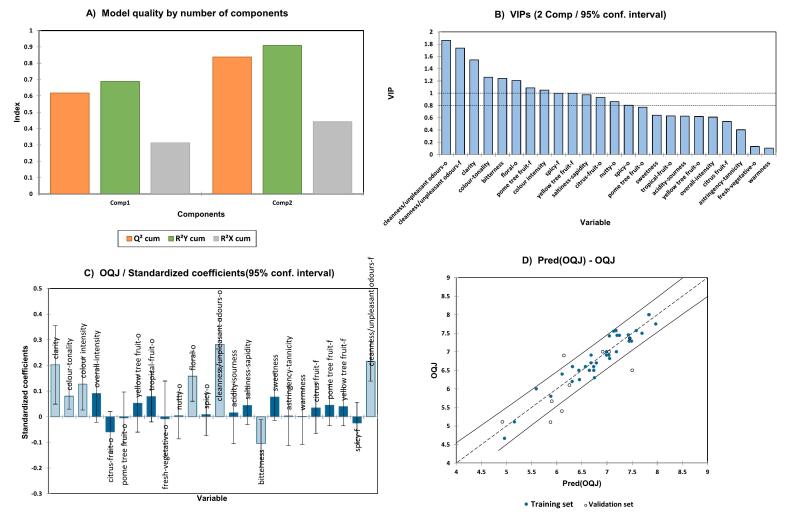
Summary of PLS regression model for the overall quality judgement over the sensory descriptors. **A**: Histogram of the model quality over two components with Q^2^, R^2^X and R^2^Y indexes, **B**: VIP for all sensory variables; **C:** effects of the variables on the PLS equation; **D**: PLS-predicted vs. experimental values of overall quality judgement.

The “overall quality judgement” is a sensory descriptor defined by the panel and is an objective assessment of all the sensory features. To study the relationship between the sensory variables and the overall quality judgement, a PLS regression was done. The sensory variables were used for regression as the PLS X-matrix (as independent variables) and the overall quality was used as the y-vector (as dependent variable). By evaluating of quality indexes for the model depending on the number of factors applied, the chosen PLS model was built on two components, showing the best performance with a Q^2^ (cum) of 0.8, a R^2^X (cum) of 0.4, and a R^2^Y (cum) of 0.9 shown in Fig. 6A. The most important variables influencing the model of the overall quality (variable importance in projection, VIP > 0.8) from Fig. 6B were “clarity”, “colour-tonality”, “bitterness”, “floral-aroma”, “pome tree fruit-flavour” and “colour intensity”. Interestingly, the most important variables were also “olfactory cleanness” and “gustatory cleanness”. The variables “clarity”, “colour-tonality”, “colour-intensity”, “floral” aroma, “olfactory cleanness”, “overall intensity”, “sweetness”, and “gustatory cleanness” showed a positive contribution in the regression model while the variables “citrus fruit” aroma, “bitterness” and “spicy” flavour showed negative effect **(**Fig. 6C). The experimental observations against the model predictions of the overall quality judgement can be seen in Fig. 6D. There were no training data outside the confidence interval (α = 95 %). However, ca. 33 % of the validation set samples were found outside the confidence interval. The full regression model is reported in the **S2-PLS1-OQJ.**

As in previous studies, a PLS regression model for the overall quality was built against the sensory descriptors (Darnal et al., 2024; Poggesi et al., 2021). This corresponds to expressing the OQJ based on the sensory attributes, i.e. ranking the sensory attributes themselves according to their relevance in defining the overall quality score. The VIP parameters would indicate the most relevant attributes in influencing the sensory quality according to the PLS model.

According to the approach taken in previous studies (Darnal et al., 2024; Poggesi et al., 2021), the sensory descriptors themselves were then applied in PLS models as dependent variables against the chemical variables used as independent variables. In analogy with those past studies, as an approximation and as a way to reduce the variable numerosity, the sensory attributes were evaluated by the modalities of perception: olfactory attributes were studied only in relation to the volatile chemical profile, while visual attributes were correlated with in-solution components (phenolic compounds, basic parameters), etc.

“Floral” aroma was the only olfactory attribute with VIP > 1 in the previously discussed PLS model for the overall quality. Hence, the “floral” aroma only was further applied as a dependent variable (y) vs. the volatile compounds used as the X-matrix, to determine which volatile compounds affected the “floral” aroma the most. For this new PLS model, the “floral” aroma had a Q^2^ (cum) of 0.4, a R^2^X (cum) of 0.4, and a R^2^Y (cum) of 0.7 with two components and considering all the volatile compounds as independent variables. The VIPs graph allowed for the observation of the variables that had the most influence on the “floral” aroma **(reported in Fig. S14 -A).** Compounds ethyl hexanoate (VII), 2-hexenal (VI), nonanal (XVI), 3-octenol (XXI), ethyl nonanoate (XXVIII), benzyl alcohol (XLIV) and phenylethyl alcohol (XLVI) showed positive contribution in the regression while compounds such as ethyl caprate (XXXIII), ethyl 9-decenoate (XXXV), ethyl dodecylate (XLI) and octanoic acid (XLIX) showed negative contributions (**Fig. S14—B**). The regression graph of the predicted olfactory variables vs. the experimentally measured olfactory variables for “floral” aroma showed 6 % training set samples were outside the confidence intervals (α = 95 %) while 42 % of the validation set was outside the 95 % confidence intervals. The whole regression model is reported in the **S2-PLS1-Floral**.

Furthermore, a PLS2 model was applied to the OQJ-VIP-filtered sensory (visual and gustatory) descriptors “clarity”, “colour tonality”, “colour intensity” and “bitterness” as the Y-matrix, and the phenolic compounds as the X-matrix. The best PLS2 regression model was calculated at 3 components with the qualitative index Q^2^ (cum) of 0.5, a R^2^X (cum) of 0.4, and a R^2^Y (cum) of 0.7. The visual attribute “clarity” showed an R^2^ of 0.8 and an RMSE of 0.6, “colour tonality” an R^2^ of 0.8 and an RMSE of 0.4, “colour intensity” an R^2^ of 0.7 and an RMSE of. 0.4 while the gustatory attribute “bitterness” showed and R^2^ of 0.6 and RMSE of 0.4. Compounds x34 (*m/z* 129 at 4.3 min), x47 (quercetin-3-*O*-glucuronide), x24 (*m/z* 143 at 22.6 min), x48 (ethyl gallate), x41 (*m/z* 464.1 at 35.5 min), x45 ((−)-epicatechin) and x23 (*m/z* 218 at 22.4 min) were the most important variables. The VIPs and the predicted vs experimental data for the different visual and gustatory attributes are shown in **Fig. S15**. Additionally, the effect of the explanatory variables on each sensory descriptor is shown in **Fig. S16**. For “clarity”, the model showed 3 % of the training set sample and ca. 33 % of the validation samples outside the confidence intervals (α = 95 %). For “colour tonality” only ca. 33 % of the validation sample was outside the confidence band. In the case of “colour intensity”, only 8 % of the validation samples were out. For “bitterness”, 2.73 % of the training set samples and 17 % of the validation samples were outside the 95 % confidence intervals. The R^2^, model standard deviation, MSE and RMSE are reported for the visual and gustatory variables used in the regression in **Table S24**. The full regression model is reported in **S2- PLS2-V&G**.

## 5 Discussion

The effect of vineyard locations on ‘Pinot Blanc’ musts and young wines was studied. Regarding the oenological parameters, it was observed that most of the parameters were similar for the must and the young wine samples from Montagna and Klaus (450 and 550 m.a.s.l.) while for samples from Aldino vineyard (800 m.a.s.l) the parameters were different. Significant differences in the volatile and the phenolic profiles were also observed among the samples from different vineyards. In the must samples, various classes of volatile compounds such as ethyl esters (ethyl acetate, ethyl butanoate, and ethyl palmitate), higher alcohols (benzyl alcohol), higher alcohol acetate such as isoamyl acetate, fatty acid degradation products (1-hexanol, 2-hexenol, hexanal, cis-3-hexenal and decanal), C_13_-norisoprenoids (β-damascenone) and other volatile phenols and benzene derivatives such as 2-phenylethyl acetate were observed. A similar group of volatile compounds was identified in the juice of different white grape varieties (Slegers et al., 2017). In the young wines, in addition to ethyl esters, C_13_-norisoprenoids, other fermentation compounds (isoamyl octanoate), and volatile phenols, volatile compounds such as terpenoids (farnesyl acetate and geranyl acetate) were also observed. Also, styrene was present in the young wines. Styrene is a petroleum-derived aromatic hydrocarbon and is known to be present in grape juice and wines as contaminant. However, styrene is structurally related to various naturally occurring molecules such as cinnamic acids, cinnamic aldehydes etc. and could be formed due to biodegradation of these substances (Baldock & Hayasaka, 2004; Steele et al., 1994). Few phenolic compounds could be identified in the musts and young wines, however there were significant differences in the profile observed in samples from different vineyards. Caftaric acid, catechin, and astilbin were found to be significantly higher in the must samples as well as young wine samples from the Aldino vineyard.

The effects of pre-fermentative grape freezing, and malolactic fermentation were also studied only in the samples from the Aldino vineyard. In the measured oenological parameters of both must and the young wines, the samples with pre-fermentative grape freezing showed a relatively higher value of the residual sugar which could be due to the initial sugar concentration being high. These samples also had lower concentrations of tartaric acid compared to the others. This lower amount in the frozen sample could be attributed to the precipitation of potassium salts during the freezing and the lower solubility of acidic salts (Celotti et al., 1999). Nitrogen sources are reported to be very important for yeast metabolism during fermentation and are known to influence the aromatic profile and the quality of wine (Fermentativa & Vinho, 2011). Alfa amino nitrogen was found to be higher in the frozen must sample while ammonia nitrogen was higher in the control must sample. Again, in the must and young wine samples, the same class of aroma compounds were observed and there were significant differences according to the pre-fermentative grape freezing in the must samples with an additional effect of both malolactic and alcoholic fermentation on the young wine samples. In the phenolic profile of the must samples, no significant effect of the pre-fermentative grape freezing was found. However, compounds such as GRP, *trans*-caftaric acid, *cis*-coutaric acid, (+)-catechin, and (−)-epicatechin were found to be higher in the control samples.

In the young wine samples, however, significant differences in the phenolic profiles were observed. PCA was performed on the bottled wines for the datasets at different storage times. The results showed that at each storage month, there was a clear separation among the different vineyards. It was interesting to observe the samples from different vineyards were correlated with different sensory, volatile, and phenolic profiles over time. At storage times 0 and 12, the overall quality was positively correlated with the wines from Aldino vineyard with the highest altitude. The wine samples from Aldino were characterized mostly by the volatile compounds ethyl acetate (I), isoamyl acetate (V), isoamyl alcohol (IX), 2-phenylethyl butyrate (XLVII), and ethyl E-11-hexadecenoate (LV), isobutanol (IV), 1-hexanol (XIII), cis-3-hexenol (XV), nonanal (XVI), 2-hexenol (XVII), acetic acid (XXII), ethyl succinate (XXXIV), phenylethyl alcohol (XLVI), and ethyl palmitate (LIV).

Ethyl acetate is known to have an ether – like odour and also contributes to “sweet” and “fruity” descriptions in wine (Fang & Qian, 2005). Isoamyl acetate is reported to have “banana” and “pear” odour characteristic while isoamyl alcohol has “alcohol” odour character (Ferreira, 2010; Styger et al., 2011). Phenylethyl alcohol is reported to have “honey”, “spicy”, “rose” and “lilac” aromas (https://www.flavornet.org/index.html, accessed on 15 April, 2024). Isobutanol has fruity, alcohol, and solvent-like odour (Styger et al., 2011). 1-hexanol is reported to contribute to “herbaceous” and “vegetal” odour (Noguerol-Pato et al., 2009). *cis*-3-Hexenol is one of the major C6-alcohols volatiles found in wine that impart leafy, cut-grass aromas (Ruiz et al., 2019). Nonanal is reported to have odour characteristic such as “citrus” and “green” (https://www.flavornet.org/index.html, accessed on 15 April, 2024). Ethyl palmitate and ethyl succinate contribute to sweet and floral notes (Shuai et al., 2020; https://www.flavornet.org/index.html, accessed on 15 April , 2024). Indeed, the sensory attributes that characterized these wines were “clarity”, “colour intensity”, “colour tonality”, both olfactory and gustatory “cleanness”, “spicy” aroma, “floral” aroma, “citrus fruit “aroma, “acidity-sourness”, “yellow tree fruit” flavour and “nutty” aroma.

These results confirm the literature finding that wines produced at high altitudes are mostly fresh, highly acidic, less alcoholic (data not shown), and with high aromatic quality (Mansour et al., 2022). Additionally, phenolic compounds that characterized the wines from Aldino vineyards were *trans-*caftaric acid, protocatechuic acid, hexoside, *trans*-caffeic acid, GRP, astilbin, (+)-catechin and gallic acid.

Furthermore, changes in sensory characteristics due to the effect of pre-fermentative grape freezing and type of inoculation were observed in the samples from the Aldino vineyard. Significant changes in attributes at different storage times were observed separately. Interestingly, “overall quality” score was always seen to be higher at the T12 storage month even though it was not a significant change. To better observe the effect of these factors on the volatile and the phenolic profiles, PCA was performed at different storage times. There was some effect of grape freezing and the type of co-inoculum in the volatile profile of these wines at different storage times. In the case of the phenolic profiles, the effect of both pre-fermentative grapes freezing, and the use of co-inoculum could be observed at T0 storage time. At T6, the effect of pre-fermentative grape freezing was more evident and finally at T12, no specific trend could be observed.

Finally, regression models to show the relationship between the overall quality judgement (OQJ) and sensory attributes have been reported. Important sensory variables for ‘Pinot Blanc’ wines were “clarity”, “colour-tonality”, “bitterness”, “floral-aroma”, “pome tree fruit-flavour”, “colour intensity”, “olfactory cleanness” and “gustatory cleanness”. Similar results have already been reported by Poggesi et al., 2021 where the most important variables were clarity, cleanness and pome tree fruits (apple, pear). These important variables were further used to build regression models to see the relationship between the aroma with the volatile compounds and between the visual and gustatory attributes with the phenolic compounds as well. For “floral” attribute, the most important volatile compounds were ethyl hexanoate (VII), 3-octenol (XIX), ethyl caprate (XXXII), benzyl alcohol (XLIV), butenolide (XXXVI), nonanal (XV), benzaldehyde (XXV), ethyl palmitoleate (XLV), isobutanol (IV), ethyl 9-decenoate (XXXV), ethyl dodecylate (XLI), tetradecanol (LII), phenylethyl alcohol (XLVI), ethyl butanoate (II), ethyl succinate (XXXIV), ethyl octanoate (XIX) and 2-phenylethyl acetate (XXXIX) while the most important variables for the visual and gustatory attributes were x34 (*m/z* 129 at 4.3 min), x47 (quercetin-3-*O*-glucuronide), x24 (*m/z* 143 at 22.6 min), x48 (ethyl gallate), x41 (*m/z* 464.1 at 35.5 min), x45 ((−)-epicatechin) and x23 (*m/z* 218 at 22.4 min).

## 6 Conclusion

In this study, differences in the sensory and the chemical profile in ‘Pinot Blanc’ musts and wines from different vineyard locations in South Tyrol (Italy) were observed. Relationships between the chemical and the sensory results have been investigated in the ‘Pinot Blanc’ wines for which not much literature can be found.

The wine samples from the highest altitude (800 m.a.s.l) were found to have the highest overall quality score, which correlated with sensory attributes such as “clarity”, “colour intensity”, “colour tonality”, both olfactory and gustatory “cleanness”, “spicy” aroma, “citrus fruit “aroma, “acidity-sourness”, “yellow tree fruit” flavour and “nutty” aroma obtained with different winemaking processes and aged in bottle for 12 months. Mountainous viticultural regions have many unique environmental conditions. These can include cool temperatures that slow down the ripening process of the grapes and allow longer growing season which in turn helps the grapes retain high acidity. This means that grapes can be harvested with optimal titratable acidity and pH levels, and acid adjustment may not be required. The cooler climate may also lead to later harvests, allowing for the development of complex flavours. Indeed, the grapes from Aldino vineyards were harvested later than the other vineyards and had higher amounts of organic acids in both must and young wines. Additionally, the bottled wines had higher “acidity/sourness” sensory attributes than the samples from other vineyards.

In addition, increased UV radiation due to the thinner atmosphere allows more intense sunlight that enhances the synthesis of phenolic compounds in grape skins, providing better colour, structure, and aging potential in wine. This was seen in must samples from Aldino which had significantly higher phenolic compounds and also the “colour intensity” of the bottled wines at all storage times.

Mountain vineyards may also have better airflow which reduces the risk of diseases such as mildew and rot, allowing for more organic and sustainable viticultural practices resulting in healthier grapes and eventually cleaner wines. The wines from Aldino always had significantly higher “clarity” and both olfactory and gustatory “cleanness” storage times 0 and 12.

Finally, mountain vineyards may promote enhanced aromatics in wines. Usually, ‘Pinot Blanc’ grown in high-altitude vineyards would be expected to exhibit vibrant “citrus”, “stone-fruit”, and “floral” notes with balanced structure and a lingering finish, and this was seen in the wines from Aldino vineyard. Consequently, new mountain sites above 1000 m.a.s.l. have been reported to be suitable for viticulture by the end of this century (Caffarra & Eccel, 2011).

Additionally, for Aldino vineyard, four different vinifications were studied, based on combinations of two key variables, pre-fermentative grape freeing and co-inoculation of lactic acid bacteria simultaneously with the yeasts. The effect of these variables was very prominent on the general oenological parameters, and also both sensory and chemical profiles. No clear effect of pre-fermentative grape freezing and type of co-inoculum were observed on the volatile profiles of these wines. Few phenolic compounds could be identified, but they were affected by both factors. The quality parameters were also studied through regression approaches in wines from different vineyards.

Growing grapes at high altitudes has shown a clear advantage in this study and these insights could not only be useful for the winemakers in replacing the conventional winemaking procedures for commercial applications but also provide a tool for wine authenticity (Kalogiouri et al., 2024). The conditions in high-altitude vineyards allow for fewer treatments with phytosanitary products and for the production of more balanced wines that do not require as much adjustment in the winery such as acidification with tartaric acid or the addition of exogenous tannins. Winemakers will need to take into account differences in harvest dates, but this may allow for a longer harvest period that can be easier to manage in the winery than a short, compressed harvest period. It is hoped that these insights will be invaluable for winemakers who practice heroic viticulture in mountainous areas, including vineyards on steep slopes and hillsides, to assist with the production of high-quality wines in the face of recent climate changes.

## Ethical Statement

The authors confirm that the appropriate protocols for protecting the rights and privacy of all participants were utilized during the execution of the research, including no coercion to participate, full disclosure of study requirements and risks, verbal consent of participants, no release of participant data without their knowledge, and the ability to withdraw from the study at any time.

## Funding

The study was funded with the project ‘Wine Identity Card’ (WineID, ID project 2019, Free University of Bolzano, number 3666) and supported by the project ‘Wine fingerprint for authenticity and improving the winemaking technology’ (Oenolab) from the 10.13039/501100008815Free University of Bozen-Bolzano (TN2810 Start-up fund). Additionally, the Autonomous Province of Bolzano, Department of Innovation, Research, and University is kindly acknowledged for its financial support for the Capacity building 1 (Decision n. 1472, 7 October 2013). This publication has been supported by the Open-Access fund from the library of the Free University of Bozen - Bolzano.

## CRediT authorship contribution statement

**Aakriti Darnal:** Writing – review & editing, Writing – original draft, Visualization, Validation, Software, Methodology, Investigation, Formal analysis, Data curation, Conceptualization. **Simone Poggesi:** Writing – review & editing, Methodology, Investigation, Formal analysis. **Vakarė Merkytė:** Methodology, Investigation, Formal analysis. **Edoardo Longo:** Writing – review & editing, Validation, Supervision, Methodology, Investigation, Formal analysis, Conceptualization. **Emanuele Boselli:** Writing – review & editing, Supervision, Resources, Project administration, Funding acquisition, Conceptualization.

## Declaration of competing interest

The authors declare that they have no known competing financial or non-financial interests or personal relationships that could have appeared to influence the work reported in this paper.

## Data Availability

Data will be made available on request.

## Appendix A Supplementary data

Supplementary material 1. Table S1; Table S2; Table S3; Table S4; Figure S1; Figure S2; Figure S3; Figure S4; Figure S5; Figure S6; Figure S7; Figure S8; Figure S9; Table S5; Figure S10; Figure S11; Figure S12; Figure S13; Table S6; Table S7; Table S8; Table S9; Table S10; Table S11; Table S12; Table S13; Table S14; Table S15; Table S16; Table S17; Table S18; Table S19; Table S20; Table S21; Table S22; Table S23; Figure S14; Figure S15; Figure S16; Table S24. Image 1

Supplementary material 2. Complete output of the PLS models. PLS1 for OQJ; PLS1 for 'floral' attribute; and PLS2 for 'visual' and 'gustatory' attributes. Image 2
