# Supplementary material for: South-Tyrolean pinot blanc identity: Exploration of chemical and sensory profile changes ascribed to vineyard locations and winemaking variables
Source: Food Chem X. 2024 Sep 10;24:101824. doi: 10.1016/j.fochx.2024.101824 (PMC11421253; doi:10.1016/j.fochx.2024.101824)
Supplement: Supplementary file 1 — Supplementary material 1. Table S1; Table S2; Table S3; Table S4; Figure S1; Figure S2; Figure S3; Figure S4; Figure S5; Figure S6; Figure S7; Figure S8; Figure S9; Table S5; Figure S10; Figure S11; Figure S12; Figure S13; Table S6; Table S7; Table S8; Table S9; Table S10; Table S11; Table S12; Table S13; Table S14; Table S15; Table S16; Table S17; Table S18; Table S19; Table S20; Table S21; Table S22; Table S23; Figure S14; Figure S15; Figure S16; Table S24. [file mmc1.docx]

**South-Tyrolean Pinot Blanc identity: Exploration of chemical and sensory profile changes ascribed to vineyard locations and winemaking variables**

Aakriti Darnal^1^, Simone Poggesi^2^, Vakarė Merkytė^1^, Edoardo Longo^1^*, Emanuele Boselli^1,2^

AFFILIATIONS

1) Oenolab, Free University of Bozen-Bolzano, Faculty of Agricultural, Environmental, and Food Sciences, Piazza Università 5, 39100 Bolzano, Italy

2) Food Experience and Sensory Testing (Feast) Lab, Massey University, Palmerston North 4410, New Zealand

3) Competence Center on Food Fermentations, Free University of Bozen-Bolzano

AUTHOR INFORMATION

*Corresponding author:

Dr. Edoardo Longo, Faculty of Agricultural, Environmental, and Food Sciences, Free University of Bozen-Bolzano, Italy; E-mail: [edoardo.longo@unibz.it](mailto:edoardo.longo@unibz.it); Phone: +39 – 0471017691; ORCID ID: <https://orcid.org/0000-0002-0594-6722>

Other authors:

Aakriti Darnal – Free University of Bozen-Bolzano; ORCID ID: <https://orcid.org/0000-0002-3834-0030>

Simone Poggesi – Food Experience and Sensory Testing (Feast) Lab, Massey University, Palmerston North, New Zealand; ORCID ID: <https://orcid.org/0000-0002-7031-3892>

Vakarė Merkytė – Free University of Bozen-Bolzano; ORCID ID: <https://orcid.org/0000-0003-2030-2041>

Emanuele Boselli – Free University of Bozen – Bolzano; ORCID ID: <https://orcid.org/0000-0001-7931-6961>

**Table S1.** Detailed description of the winemaking procedure for each vineyard. MM=Montagna, K = Klaus, V= Aldino, C= Control, LL= Co-inoculation with selected liquid yeast and lactic acid bacteria, F= Frozen grapes, NF= Non-frozen grapes.

| **Variant** | **Description** |
| --- | --- |
| **MM_C** | Grapes were crushed and pressed one day after they were harvested. Pectolytic enzymes were added (3mL^.^hL^-1^) to accelerate must clarification and enhance aroma extraction (Everzym, from Ever, Pramaggiore, Italy). Oxygen treatment of the lower turbid part of the hard-pressed juice for 12 hours at room temperature and filtered, then added back to the lightly pressed juice. The juice was inoculated with Crublanc Maurivin and La Fuitee Oeno (50:50). Lysopol (vitamin B) and ammonium salts (5 g of (NH_4_)_2_HPO_4_) as nitrogen source and yeast stimulation were added. The fermentation then lasted for nine days before the wine was treated with 3g^.^hL^-1^ SO_2_ (sulfur dioxide) and 3 g^.^hL^-1^ oak+chestnut tannin before getting racked into a barrel. |
| **K_C** | The grapes were crushed the day they were harvested. 5 g^.^hL^-1^ SO_2_ and 5 g^.^hL^-1^ oak tannins were added to the lightly pressed must (0.5 bar), and static clarification was employed for 12 hours. The hard-pressed juice received an acid correction (7 gL^-1^ of tartaric acid) and oxidation but was withheld from being added to the light-press juice. Crublanc Maurivin was used for inoculation. The fermentation lasted for twelve days after which the wines were racked in a stainless-steel tank for aging. |
| **V_C / V_NF_C** | The control was pressed at 0.5 bar (soft-pressed) and 1.4 bar (hard-pressed) juice was collected, filtered, and oxidized before returning to the lightly pressed juice. Crublanc Maurivin yeast was used to inoculate this tank, and the fermentation took six days before it was racked into barrels for aging. |
| **V_NF_LL** | Same pressing, juice consolidation, and aging protocol as V_C. However, this was inoculated with a lactic acid bacteria culture with selected liquid yeast causing sequential alcoholic and malolactic fermentation. |
| **V_F_C** | The grapes were frozen for seven days at -20°C and were crushed after thawing. The pressing process was the same as mentioned above for V_C. However, the hard press juice was treated with gelatin to remove unwanted phenolics. The fermentation with Crublanc Maurivin yeast took six days before the wine was racked into barrels. |
| **V_F_LL** | The grapes were frozen for seven days at a temperature of -20°C. The same pressing protocol was used as mentioned above. However, this was co-inoculated with lactic acid bacteria and selected liquid yeast for sequential malolactic and alcoholic fermentation. |

**Table S2.** Target Analyte Finding Parameters.

| Analyte | Tolerance | Units | Start Time | End Time | Start 2nd Dim time | End 2nd dim time | Min Area | Min Height | Signal Mode |
| --- | --- | --- | --- | --- | --- | --- | --- | --- | --- |
| Ethyl acetate | 0.3 | Da | 5.1 min | 6.6 min | 0.500 s | 1.100 s | 1 | 1 | Profile |
| Butanoic acid, ethyl ester | 0.3 | Da | 8.3 min | 11.7 min | 1.500 s | 1.840 s | 1 | 1 | Profile |
| 1-Propanol,2-methyl- | 0.3 | Da | 10.3 min | 11.8 min | 0.400 s | 0.900 s | 1 | 1 | Profile |
| Hexanal | 0.3 | Da | 11.1 min | 12.5 min | 1.430 s | 1.550 s | 1 | 1 | Profile |
| 1-Butanol, 3-methyl-, acetate | 0.3 | Da | 11.8 min | 13.5 min | 1.200 s | 2.100 s | 1 | 1 | Profile |
| 2-methyl-3-pentanol (IS) | 0.3 | Da | 14.6 min | 16.7 min | 1.000 s | 1.400 s | 1 | 1 | Profile |
| 1-butanol, 3-methyl- | 0.3 | Da | 15.8 min | 17.8 min | 0.500 s | 0.900 s | 1 | 1 | Profile |
| hexanoic acid, ethyl ester | 0.3 | Da | 17 min | 17.8 min | 1.500 s | 2.000 s | 1 | 1 | Profile |
| 2-Hexenal | 0.3 | Da | 17.5 min | 17.8 min | 1.000 s | 1.600 s | 1 | 1 | Profile |
| 3-Hexenoic acid, ethyl ester | 0.3 | Da | 18.3 min | 20 min | 1.800 s | 2.200 s | 1 | 1 | Profile |
| Styrene | 0.3 | Da | 18.9 min | 19.5 min | 1.00 s | 1.700 s | 1 | 1 | Profile |
| acetic acid, hexyl ester | 0.3 | Da | 19.7 min | 21.2 min | 1.600 s | 2.150 s | 1 | 1 | Profile |
| 3-Hexen-1-ol, acetate, (Z)- | 0.3 | Da | 21.6 min | 22.7 min | 1.500 s | 1.900 s | 1 | 1 | Profile |
| 1-Hexanol | 0.3 | Da | 22.8 min | 23 min | 0.700 s | 1.500 s | 1 | 1 | Profile |
| 4-mercapto-4-methyl-2-pentanone | 0.3 | Da | 24.5 min | 25.2 min | 1.185 s | 1.312 s | 1 | 1 | Profile |
| Nonanal | 0.3 | Da | 25.1 min | 26.1 min | 1.650 s | 1.950 s | 1 | 1 | Profile |
| octanoic acid, ethyl ester | 0.3 | Da | 25.5 min | 26.6 min | 1.400 s | 2.100 s | 1 | 1 | Profile |
| 3-Hexen-1-ol, (Z)- | 0.3 | Da | 25.6 min | 26 min | 0.900 s | 1.400 s | 1 | 1 | Profile |
| 2-Hexen-1-ol | 0.3 | Da | 26.1 min | 26.8 min | 0.970 s | 1.100 s | 1 | 1 | Profile |
| 2-isopropyl-3-methoxypyrazine | 0.3 | Da | 26.8 min | 27.5 min | 1.558 s | 1.828 s | 1 | 1 | Profile |
| furfuryl mercaptan | 0.3 | Da | 26.8 min | 27.4 min | 1.032 s | 1.185 s | 1 | 1 | Profile |
| Furfural | 0.3 | Da | 27.2 min | 27.5 min | 0.400 s | 0.800 s | 1 | 1 | Profile |
| 1-octen-3-ol | 0.3 | Da | 27.9 min | 28.5 min | 1.125 s | 1.292 s | 1 | 1 | Profile |
| Isopentyl hexanoate | 0.3 | Da | 28.1 min | 28.7 min | 2.500 s | 2.800 s | 1 | 1 | Profile |
| Acetic acid | 0.3 | Da | 28.2 min | 29 min | 0.700 s | 1.000 s | 1 | 1 | Profile |
| Acetic acid, octyl ester | 0.3 | Da | 28.9 min | 29.3 min | 2.000 s | 2.200 s | 1 | 1 | Profile |
| 2-sec-buthyl-3-methoxypyrazine | 0.3 | Da | 29.7 min | 30.4 min | 1.671 s | 1.884 s | 1 | 1 | Profile |
| Decanal | 0.3 | Da | 29.7 min | 30.7 min | 1.700 s | 2.100 s | 1 | 1 | Profile |
| 1-Hexanol, 2-ethyl | 0.3 | Da | 29.7 min | 30.4 min | 1.000 s | 1.400 s | 1 | 1 | Profile |
| Benzaldehyde | 0.3 | Da | 30 min | 30.8 min | 0.800 s | 1.300 s | 1 | 1 | Profile |
| 2,3-Butanediol | 0.3 | Da | 31 min | 35 min | 0.500 s | 0.600 s | 1 | 1 | Profile |
| 2-Nonanol | 0.3 | Da | 31 min | 31.7 min | 1.100 s | 1.550 s | 1 | 1 | Profile |
| Nonanoic acid, ethyl ester | 0.3 | Da | 31 min | 32.2 min | 1.800 s | 2.900 s | 1 | 1 | Profile |
| trans-2-nonenal | 0.3 | Da | 31.1 min | 31.7 min | 1.465 s | 1.645 s | 1 | 1 | Profile |
| linalool | 0.3 | Da | 32 min | 32.4 min | 1.245 s | 1.484 s | 1 | 1 | Profile |
| 1-Octanol | 0.3 | Da | 32.2 min | 33.4 min | 0.800 s | 1.600 s | 1 | 1 | Profile |
| 1-Butanol | 0.3 | Da | 32.6 min | 32.8 min | 1.000 s | 1.400 s | 1 | 1 | Profile |
| decanoic acid, ethyl ester | 0.3 | Da | 33.3 min | 35 min | 1.500 s | 1.900 s | 1 | 1 | Profile |
| 2-Furancarboxylic acid, ethyl ester | 0.3 | Da | 34.8 min | 35.2 min | 0.900 s | 1.200 s | 1 | 1 | Profile |
| butanedioic acid, diethyl ester | 0.3 | Da | 35.6 min | 36.3 min | 0.900 s | 1.200 s | 1 | 1 | Profile |
| Ethyl 9 -decenoate | 0.3 | Da | 36 min | 36.8 min | 1.300 s | 1.800 s | 1 | 1 | Profile |
| octanoic acid, 3-methylbutyl ester | 0.3 | Da | 36.2 min | 37.1 min | 2.600 s | 3.400 s | 1 | 1 | Profile |
| α-Terpineol | 0.3 | Da | 36.5 min | 37 min | 0.800 s | 1.100 s | 1 | 1 | Profile |
| α-Calacorene | 0.3 | Da | 37.6 min | 37.8 min | 1.300 s | 1.800 s | 1 | 1 | Profile |
| 3-mercapto-hexyl acetate | 0.3 | Da | 38.2 min | 39 min | 1.230 s | 1.550 s | 1 | 1 | Profile |
| 1H-indene, 1-methylene- | 0.3 | Da | 38.8 min | 39.5 min | 1.000 s | 1.400 s | 1 | 1 | Profile |
| 1-Penten-3-one | 0.3 | Da | 39.7 min | 40.2 min | 0.300 s | 1.400 s | 1 | 1 | Profile |
| Geranyl acetate | 0.3 | Da | 39.7 min | 40.4 min | 1.400 s | 1.900 s | 1 | 1 | Profile |
| methyl salicylate | 0.3 | Da | 40.1 min | 40.76 min | 0.800 s | 1.600 s | 1 | 1 | Profile |
| 1-Decanol | 0.3 | Da | 40.2 min | 40.85 min | 1.150 s | 1.400 s | 1 | 1 | Profile |
| p-cumenol | 0.3 | Da | 40.3 min | 40.5 min | 0.800 s | 1.200 s | 1 | 1 | Profile |
| Acetic acid, 2-phenylethylester | 0.3 | Da | 40.5 min | 41 min | 0.900 s | 1.500 s | 1 | 1 | Profile |
| Dodecanoic acid, ethyl ester | 0.3 | Da | 41.5 min | 42.3 min | 1.500 s | 2.100 s | 1 | 1 | Profile |
| β-Damascenone | 0.3 | Da | 42 min | 42.8 min | 1.300 s | 1.600 s | 1 | 1 | Profile |
| 3-mercapto-hexanol | 0.3 | Da | 43.1 min | 43.4 min | 0.850 s | 1.100 s | 1 | 1 | Profile |
| 5,9-Undecadien-2-one, 6,10-dimethyl- | 0.3 | Da | 43.4 min | 43.9 min | 1.450 s | 1.850 s | 1 | 1 | Profile |
| Pentadecanoic acid, 3-methylbutyl ester | 0.3 | Da | 43.5 min | 44.3 min | 2.700 s | 3.400 | 1 | 1 | Profile |
| Phenylethyl alcohol | 0.3 | Da | 44 min | 44.7 min | 0.500 s | 0.800 s | 1 | 1 | Profile |
| 2(3H)-Furanone,5-butyldihydro-4-methyl-,cis | 0.3 | Da | 44.3 min | 45.5 min | 0.800 s | 1.600 s | 1 | 1 | Profile |
| Benzyl alcohol | 0.3 | Da | 44.4 min | 45 min | 0.700 s | 1.500 s | 1 | 1 | Profile |
| Undecanoic acid, ethyl ester | 0.3 | Da | 44.5 min | 45.7 min | 1.700 s | 2.700 s | 1 | 1 | Profile |
| β-Phenylethyl butyrate | 0.3 | Da | 47 min | 47.5 min | 1.200 s | 1.400 s | 1 | 1 | Profile |
| Phenol | 0.3 | Da | 48.4 min | 49.1 min | 0.500 s | 1.000 s | 1 | 1 | Profile |
| Octanoic acid | 0.3 | Da | 48.8 min | 49.3 min | 0.460 s | 0.700 s | 1 | 1 | Profile |
| 4-ethylguaiacol | 0.3 | Da | 49 min | 49.5 min | 0.946 s | 1.119 s | 1 | 1 | Profile |
| nerolidol | 0.3 | Da | 49.6 min | 50.1 min | 1.478 s | 1.677 s | 1 | 1 | Profile |
| Triacetin | 0.3 | Da | 50.9 min | 51.5 min | 0.850 s | 1.200 s | 1 | 1 | Profile |
| ethyl-cynnamate | 0.3 | Da | 52.1 min | 52.7 min | 1.072 s | 1.312 s | 1 | 1 | Profile |
| 1-Dodecanol | 0.3 | Da | 53.8 min | 54.1 min | 1.100 s | 1.600 s | 1 | 1 | Profile |
| Phenol,4-ethyl | 0.3 | Da | 53.9 min | 54.3 min | 0.600 s | 1.010 s | 1 | 1 | Profile |
| hexadecanoic acid, ethyl ester | 0.3 | Da | 54.1 min | 54.7 min | 1.200 s | 1.700 s | 1 | 1 | Profile |
| 2,6,10-Dodecatrien-1-ol, 3,7,11-trimethyl-, acetate, (E,E)- | 0.3 | Da | 55.1 min | 56 min | 1.100 s | 1.570 s | 1 | 1 | Profile |
| Ethyl-9-hexadecanoate | 0.3 | Da | 55.6 min | 56.3 min | 1.400 s | 1.800 s | 1 | 1 | Profile |
| octadecanoic acid, ethyl ester | 0.3 | Da | 58.2 min | 58.6 min | 1.450 s | 1.700 s | 1 | 1 | Profile |
| Ethyl 9-octadecanoate | 0.3 | Da | 58.6 min | 59 min | 1.350 s | 1.650 s | 1 | 1 | Profile |

**Table S3.** List of descriptors and respective definitions of the attributes for Pinot Blanc.

| **Attribute** | **Descriptors** | **Description** | **Attribute** |
| --- | --- | --- | --- |
| **VISUAL** | Clarity | Absence of veiling, suspensions (precipitating chemical compounds), and solid particles (precipitates, cork pieces). | |
|  | Colour tonality | From straw with a greenish hue to golden yellow | Look in between the core and rim and choose the scale. |
|  | Colour intensity |  |  |
| **OLFACTORY** | Overall intensity | Taking into consideration all aroma profiles. | |
|  | Citrus fruit | Lemon, grapefruit | An aroma of the lemon zest or juices of lemon and grapefruit. |
|  | Pome tree fruit | Apple, pear | An aroma that is typical to the pulp of pome. |
|  | Yellow tree fruit | Apricot, peach | An aroma that is typical to the yellow tree fruits. |
|  | Tropical fruit | Banana, pineapple | An aroma of fruit that is tropical. |
|  | Fresh vegetative | Bell pepper, grass | A vegetative aroma that is typical to fresh greens (e.g. freshly sliced bell pepper and freshly cut grass). |
|  | Nutty | Almond, walnut | An aroma that can be described by almond, walnut or marzipan. |
|  | Floral | Wildflowers, jasmine, rose | A fresh scent of flowers, typically found in the countryside and garden: jasmine, rose. |
|  | Spicy | White pepper, nutmeg (noce moscata) | An aroma of white pepper, nutmeg (noce moscata). |
|  | Cleanness | Absence of unpleasant odours (faults and taints). | |
| **GUSTATORY** (taste, chemesthetic sensations and flavours) | Acidity/Sourness | Acid | Having an acidic taste, that resembles vinegar, lemon juice. |
|  | Saltiness/Sapidity | Salt/Glutamate | Having a salty/savoury taste. |
|  | Bitterness | Caffeine ̶̶ > coffee | Having a bitter taste, that resembles coffee, tea, cacao. |
|  | Sweetness | Sugar | Having a sweet taste that comes from sugars. |
|  | Astringency/tannicity | Tannins, alum | Puckering, drying mouthfeel (e.g. if you´d eat unripe cachi fruits). |
|  | Warmness | Alcohol | Warm sensation in the mouth. |
|  | Citrus fruit | Lemon, grapefruit | Having a flavour that is typical to citrus fruits. |
|  | Pome tree fruit | Apple, pear | Having a flavour that is typical to pome tree fruits. |
|  | Yellow tree fruit | Apricot, peach | Having a flavour that is typical to yellow tree fruits. |
|  | Spicy | White pepper, nutmeg (noce moscata) | Having a flavour that resembles white pepper, nutmeg (noce moscata). |
|  | Cleanness | Absence of unpleasant flavours (faults and taints). | |
| **Overall quality judgement** | | An objective answer about the quality of wine, not linked to your personal liking. | |

**Table S4.** List of identified volatile compounds in Pinot Blanc samples.

| Number | Name | RT I (min) | RT II (sec) | Retention Index (RI) | Base mass (m/z) |
| --- | --- | --- | --- | --- | --- |
| I | Ethyl acetate | 5.7 | 1.0 | 959 | 43 |
| II | Ethyl butanoate | 10.1 | 1.7 | 1065 | 71 |
| III | Hexanal | 11.8 | 1.5 | 1104 | 44 |
| IV | Isobutanol | 12.9 | 0.8 | 1131 | 43 |
| V | Isoamyl acetate | 13.4 | 1.7 | 1143 | 43 |
| VI | 2-Hexenal | 17.8 | 1.3 | 1245 | 41, 55 |
| VII | Ethyl hexanoate | 18.4 | 2.1 | 1258 | 43, 88 |
| VIII | Ethyl trans-hex-3-enoate | 18.7 | 1.9 | 1266 | 41 |
| IX | Isoamyl alcohol | 19.1 | 1.1 | 1275 | 41, 55 |
| X | Styrene | 19.2 | 1.4 | 1261 | 78 |
| XI | Hexyl acetate | 20.3 | 2.0 | 1302 | 43 |
| XII | (E)-3-Hexenyl acetate | 22.7 | 1.8 | 1354 | 41 |
| XIII | 1-Hexanol | 23.9 | 1.1 | 1382 | 56 |
| XIV | n/a | 25.1 | 1.2 | / | 43 |
| XV | cis-3-Hexenol | 25.8 | 1.1 | 1424 | 41,67 |
| XVI | Nonanal | 25.9 | 1.8 | 1427 | 44 |
| XVII | 2-hexenol | 26.3 | 1.2 | 1435 | 41, 71 |
| XVIII | n/a | 27.2 | 1.1 | / | 53, 81 |
| XIX | Ethyl octoate | 27.5 | 2.3 | 1419 | 88 |
| XX | Isopentyl hexanoate | 28.2 | 2.6 | 1481 | 43,70 |
| XXI | 3-Octenol | 28.2 | 1.2 | 1482 | 57 |
| XXII | Acetic acid | 28.5 | 0.8 | 1488 | 43 |
| XXIII | n/a | 29.1 | 2.2 | / | 43 |
| XXIV | Ethyl hexanol | 29.9 | 1.3 | 1476 | 41 |
| XXV | n/a | 30.1 | 2.0 | / | 44 |
| XXVI | Benzaldehyde | 30.6 | 1.1 | 1540 | 51 |
| XXVII | n/a | 31.3 | 1.5 | / | 41 |
| XXVIII | Ethyl nonanoate | 31.5 | 2.4 | 1562 | 88 |
| XXIX | 2,3-Butanediol | 31.6 | 0.8 | 1565 | 45 |
| XXX | Linalool | 32.2 | 1.3 | 1581 | 41, 71 |
| XXXI | n-Octanol | 32.7 | 1.0 | 1605 | 43 |
| XXXII | Isoamyl octanoate | 36.3 | 2.6 | 1675 | 43 |
| XXXIII | Ethyl caprate | 36.7 | 2.2 | 1683 | 88 |
| XXXIV | Ethyl succinate | 37.5 | 1.2 | 1699 | 55, 101 |
| XXXV | Ethyl 9 -decenoate | 37.7 | 2.1 | 1710 | 88 |
| XXXVI | Butenolide | 40.0 | 0.8 | 1753 | 55,84 |
| XXXVII | Geranyl acetate | 40.0 | 1.7 | 1754 | 44, 69 |
| XXXVIII | 1-Decanol | 40.7 | 1.2 | 1767 | 41 |
| XXXIX | 2-Phenylethyl acetate | 42.3 | 1.2 | 1801 | 43, 104 |
| XL | β-Damascenone | 42.3 | 1.4 | 1802 | 69 |
| XLI | Ethyl dodecylate | 43.4 | 2.5 | 1825 | 88 |
| XLII | Geraniol | 43.5 | 1.8 | 1827 | 41 |
| XLIII | Isoamyl decanoate | 43.7 | 2.6 | 1831 | 43 |
| XLIV | Benzyl alcohol | 44.7 | 0.9 | 1865 | 79 |
| XLV | ester (n/a) | 45.4 | 2.4 | 1899 | 88 |
| XLVI | Phenylethyl alcohol | 46.0 | 0.9 | 1927 | 91 |
| XLVII | 2-Phenylethyl butyrate | 47.3 | 1.3 | 1989 | 71, 104 |
| XLVIII | Phenol | 47.2 | 0.5 | 1985 | 39, 66 |
| XLIX | Octanoic acid | 50.5 | 1.4 | 2145 | 60, 94 |
| L | n/a | 51.2 | 1.0 | / | 43 |
| LI | n/a | 52.4 | 1.2 | / | 77, 131 |
| LII | Tetradecanol | 54.1 | 1.4 | 2315 | 43 |
| LIII | Farnesyl acetate | 55.6 | 1.4 | 2387 | 43 |
| LIV | Ethyl palmitate | 55.6 | 1.8 | 2388 | 88 |
| LV | Ethyl E-11-hexadecenoate | 56.0 | 1.6 | 2406 | 55 |
| LVI | Ethyl palmitoleate | 58.7 | 1.5 | 2538 | 55 |

|  |
| --- |

**Figure S1.** Fingerprint GCxGC-ToF/MS retention time region; analytical-ion chromatogram for a PB must – example. Note: in the figure, only the most informative portion of the chromatogram is shown.

|  |
| --- |

**Figure S2.** GCxGC-ToF/MS analytical-ion chromatogram for a PB wine – example.

|  |
| --- |

**Figure S3.** Example of the GCxGC-ToF/MS spectra for ethyl acetate along with the workflow of processing (from raw data to subtracted and reconstructed spectrum). The three elements are Caliper which is the raw data, spectrum after deconvolution (peak true) and the library spectra (comparison between peak true and the best match in the library).

|  |
| --- |

**Figure S4.** Example of the GCxGC-ToF/MS spectra for 3-methyl-1-butyl acetate.

|  |
| --- |

**Figure S5.** Example of the GCxGC-ToF/MS spectra for hexyl acetate.

|  |
| --- |

**Figure S6.** Example of the GCxGC-ToF/MS spectra for 1-hexanol.

|  |
| --- |

**Figure S7.** Example of the GCxGC-ToF/MS spectra for linalool.

|  |
| --- |

**Figure S8.** Example of the GCxGC-ToF/MS spectra for acetic acid-2-phenylethyl ester.

|  |
| --- |

**Figure S9.** Example of the GCxGC-ToF/MS spectra for ethyl 9-decenoate.

**Table S5**. List of tentatively identified phenolic compounds in Pinot Blanc based on retention time. The compounds followed by an asterisk were assigned by observing the mass fragmentation, whereas all the other compounds were identified by injecting the related standard compounds.

| **Code** | **Compound assignment** | **R.T. (min)** | **ESI- mass**  **(*m/z*)** | **MS^2^ fragments (*m/z* )** | **UV-Vis λ_max_**  **(**nm**)** |
| --- | --- | --- | --- | --- | --- |
| x17 | myricetin* | 12.8 | 317 | 151, 287 ← 317 | too small |
| x18 | gallic acid, hexoside (*) | 13.2 | 331 |  | 267 |
| x20 | gallic acid | 17.5 | 169 |  | too small |
| x27 | protocatechuic acid, hexoside (*) | 20.7 | 315 | 153←315 | too small |
| x29 | S-glutathionylcaftaric acid (GRP) | 24.8 | 616 |  | 327 |
| x30 | *cis*-caftaric acid | 26.3 | 311 |  |  |
| x33 | *trans*-caftaric acid | 28.2 | 311 | 135, 179←311 | 297, 327 |
| x38 | *cis*-coutaric acid | 33.3 | 295 | 163←295 | 308 |
| x40 | (+)-catechin | 34.0 | 289 |  | 280 |
| x42 | *trans*-caffeic acid | 36.6 | 179 |  | 290, 330 |
| x45 | (-)-epicatechin | 38.5 | 289 |  | 279 |
| x47 | quercetin-3-O-glucuronide* | 42.1 | 477 | 301←477 | overlapping species / 355 |
| x48 | ethyl gallate* | 45.1 | 197 | 169←197 |  |
| x52 | astilbin | 50.0 | 449 |  | 290 |

|  |
| --- |

**Figure S10.** Total-Ion chromatogram and aligned PDA (280 nm) trace of a Pinot Blanc wine as an example of data obtained with LC-DAD-QqQ/MS (Full Scan, ESI-) analysis. TIC retention times have been aligned post-acquisition to the PDA retention times by checking the retention times of reference compounds in both traces.

|  |
| --- |

**Figure S11**. Example of ESI(-) full scan – MS signal and the related PDA spectra of gallic acid (*m/z* 169).

|  |
| --- |

**Figure S12**. Example of ESI(-) full scan – MS signal and the related PDA spectra of *trans*-caftaric acid (*m/z* 311 – also showing *m/z* 623 dimer adduct).

|  |
| --- |

**Figure S13**. Example of ESI(-) full scan – MS signal and the related PDA spectra of taxifolin-3-*O*-rhamnoside (astilbin, *m/z* 449– also showing *m/z* 512 adduct).

**Table S6.** Oenological parameters of musts before inoculation. M: Montagna vineyard; K: Klaus vineyard; V=Aldino vineyard.

| Vineyard | Glucose-Fructose (g^.^L^-1^ ) | Tartaric acid (g^.^L^-1^) | Acetic acid (g^.^L^-1^) | Malic acid (g^.^L^-1^ ) | Lactic acid (g^.^L^-1^) | Alfa amino nitrogen (mg^.^L^-1^) | Ammonia nitrogen (mg^.^L^-1^) |
| --- | --- | --- | --- | --- | --- | --- | --- |
| MM_C | 149.51 ± 0.01 | 2.67 ± 0.01 | 0.15 ± 0.01 | 3.43 ± 0.01 | 0.02 ± 0.01 | 233 ± 1 | 92 ± 1 |
| K_C | 150.30 ± 0.01 | 2.82 ± 0.01 | 0.19 ± 0.01 | 3.07 ± 0.01 | 0.08 ± 0.01 | 246 ± 1 | 84 ± 1 |
| V_C | 178.94 ± 0.01 | 1.92 ± 0.01 | **** | 3.79 ± 0.01 | 0.09 ± 0.01 | 137 ± 1 | **** |

The cells marked with “****” indicate values below the quantitation threshold

**Table S7.** Oenological parameters of young wines. M: Montagna vineyard; K: Klaus vineyard; V=Aldino vineyard.

| Vineyard | Glucose-Fructose (g^.^L^-1^ ) | Tartaric acid (g^.^L^-1^ ) | Acetic acid  (g^.^L^-1^ ) | Malic acid (g^.^L^-1^ ) | Lactic acid (g^.^L^-1^ ) | Total SO_2_ (mg^.^L^-1^ ) | Free SO_2_ (mg^.^L^-1^ ) | Total polyphenos (mg^.^L^-1^ ) |
| --- | --- | --- | --- | --- | --- | --- | --- | --- |
| MM_C | **** | 1.88 ± 0.01 | 0.19 ± 0.01 | 2.06 ± 0.01 | **** | 53.00 ± 1 | 12.00 ± 1 | 328.00 ± 1 |
| K_C | **** | 1.95 ± 0.01 | 0.18 ± 0.01 | 1.96 ± 0.01 | **** | 76.00 ± 1 | 11.00 ± 1 | 331.00 ± 1 |
| V_C | **** | 2.69 ± 0.01 | 0.31 ± 0.01 | 3.71 ± 0.01 | **** | 60.00 ± 1 | 13.00 ± 1 | 445.05 ± 1 |

The cells marked with “****” indicate values below the quantitation threshold

**Table S8**. One-way ANOVA of the volatile compounds present in the must samples of different vineyards.

| **Category** | **I** | **II** | **III** | **V** | **VI** | **IX** | **XIII** | **XV** |
| --- | --- | --- | --- | --- | --- | --- | --- | --- |
| **Aldino** | 11823873.667 b | 4935945.333 a | 17143279.000 a | 3076046.000 a | 60987701.000 a | 4291497.667 b | 76644925.000 b | 5504914.333 a |
| **Montagna** | 18845164.333 a | 2218935.667 b | 1228203.667 b | 1998138.667 b | 6432536.000 b | 6606626.667 a | 282001138.667 a | 2189946.000 b |
| **Klaus** | 12397555.667 b | 1516076.667 b | 437056.333 b | 1659234.667 b | 2209232.333 b | 452655.667 c | 57531308.667 b | 354759.667 b |
| **Pr > F(Model)** | 0.016 | <0.0001 | 0.001 | 0.020 | <0.0001 | 0.001 | 0.000 | 0.006 |
| **Significant** | Yes | Yes | Yes | Yes | Yes | Yes | Yes | Yes |

| **Category** | **XVII** | **XXVI** | **XXIX** | **XXXIX** | **XL** | **XLIV** | **XLV** | **LIV** |
| --- | --- | --- | --- | --- | --- | --- | --- | --- |
| **Aldino** | 44032298.333 b | 2837707.333 a | 16585860.000 a | 1399184.333 a | 1906867.333 a | 1548570.667 a | 616794.000 a | 903145.000 a |
| **Montagna** | 83772973.333 a | 2453108.333 a | 17619065.333 a | 1463944.667 a | 1187012.333 b | 1837222.333 a | 392122.667 ab | 1108745.333 a |
| **Klaus** | 17219828.000 c | 1135488.000 b | 1086650.000 b | 75955.333 b | 659736.667 c | 334913.000 b | 233441.667 b | 0.000 b |
| **Pr > F(Model)** | <0.0001 | 0.000 | <0.0001 | 0.047 | <0.0001 | 0.003 | 0.020 | 0.002 |
| **Significant** | Yes | Yes | Yes | Yes | Yes | Yes | Yes | Yes |

**Table S9**. One-way ANOVA of the volatile compounds present in the young wine samples of different vineyards.

| **Category** | **X** | **XIV** | **XXXII** | **XXXIII** | **XXXV** | **XXXVII** | **XXXIX** |
| --- | --- | --- | --- | --- | --- | --- | --- |
| **Montagna** | 82554226.667 a | 184855.667 b | 828663951.333 b | 5593794841.333 b | 88410057.667 a | 2673688.333 a | 234539592.667 a |
| **Klaus** | 41928751.667 b | 470660.667 a | 2096579645.333 a | 8172295371.333 a | 8580975.667 b | 2084267.667 ab | 124323961.000 b |
| **Aldino** | 97809441.333 a | 294787.667 b | 1073478891.667 b | 4708252675.333 b | 56600675.667 a | 1559723.333 b | 223846895.333 a |
| **Pr > F(Model)** | 0.002 | 0.006 | 0.004 | 0.027 | 0.003 | 0.036 | 0.002 |
| **Significant** | Yes | Yes | Yes | Yes | Yes | Yes | Yes |

| **Category** | **XL** | **XLI** | **XLV** | **LII** | **LIII** | **LV** |
| --- | --- | --- | --- | --- | --- | --- |
| **Montagna** | 4087034.000 a | 583790316.667 b | 1627695.333 b | 1533405.667 b | 1653112.333 b | 11727943.333 b |
| **Klaus** | 3063974.333 b | 1027765142.667 a | 3401559.667 a | 2227756.667 a | 2173437.333 a | 14196082.333 a |
| **Aldino** | 3174290.333 b | 512184138.667 b | 1546850.667 b | 997826.667 b | 1350426.667 b | 10611681.333 b |
| **Pr > F(Model)** | 0.045 | 0.006 | 0.006 | 0.011 | 0.015 | 0.006 |
| **Significant** | Yes | Yes | Yes | Yes | Yes | Yes |

**Table S10**. One-way ANOVA of the phenolic compounds present in the musts of different vineyards.

| **Category** | **x15** | **x33** | **x40** | **x52** |
| --- | --- | --- | --- | --- |
| **Aldino** | 48313.333 a | 782686.333 a | 95422.000 a | 36299.333 a |
| **Klaus** | 50830.000 a | 389886.500 b | 44368.500 b | 17030.500 b |
| **Montagna** | 18290.500 b | 114500.000 b | 15772.500 b | 18844.000 b |
| **Pr > F(Model)** | 0.032 | 0.008 | 0.011 | 0.048 |
| **Significant** | Yes | Yes | Yes | Yes |

**Table S11**. One-way ANOVA of the phenolic compounds present in the young wine samples of different vineyards.

| **Category** | **x3** | **x8** | **x15** | **x16** | **x19** | **x20** | **x23** | **x24** | **x25** |
| --- | --- | --- | --- | --- | --- | --- | --- | --- | --- |
| **Aldino** | 27834.333 b | 57014.667 ab | 8865.000 b | 140712.667 a | 60605.333 b | 25773.333 b | 15275.667 a | 18290.000 b | 14011.333 ab |
| **Klaus** | 37203.778 a | 114020.111 a | 22620.111 a | 74914.000 b | 73048.667 b | 94730.000 b | 10446.111 b | 27571.000 ab | 19950.333 a |
| **Montagna** | 33586.750 ab | 21140.000 b | 12702.250 b | 131586.000 a | 177556.375 a | 255130.375 a | 14656.000 a | 31642.750 a | 12467.000 b |
| **Pr > F(Model)** | 0.038 | 0.002 | <0.0001 | 0.003 | 0.004 | <0.0001 | 0.007 | 0.045 | 0.009 |
| **Significant** | Yes | Yes | Yes | Yes | Yes | Yes | Yes | Yes | Yes |

| **Category** | **x26** | **x27** | **x28** | **x30** | **x33** | **x34** | **x36** | **x37** | **x39** |
| --- | --- | --- | --- | --- | --- | --- | --- | --- | --- |
| **Aldino** | 21033.667 b | 45660.000 a | 21762.667 a | 34760.000 a | 725374.333 a | 42602.667 a | 107404.667 a | 102028.667 a | 13005.333 a |
| **Klaus** | 24566.111 ab | 22033.667 b | 13714.333 b | 30582.333 a | 405293.333 b | 42991.111 a | 64894.444 c | 56409.333 b | 7501.444 b |
| **Montagna** | 28101.250 a | 20890.000 b | 14861.625 b | 15812.500 b | 286557.750 c | 28506.750 b | 78041.750 b | 45307.375 b | 8232.375 b |
| **Pr > F(Model)** | 0.040 | <0.0001 | 0.039 | <0.0001 | <0.0001 | 0.000 | 0.000 | <0.0001 | 0.001 |
| **Significant** | Yes | Yes | Yes | Yes | Yes | Yes | Yes | Yes | Yes |

| **Category** | **x40** | **x41** | **x42** | **x43** | **x45** | **x47** | **x48** | **x52** |
| --- | --- | --- | --- | --- | --- | --- | --- | --- |
| **Aldino** | 68467.667 a | 10789.000 a | 19687.333 a | 27830.333 b | 24038.333 a | 33373.333 b | 67513.000 a | 66629.333 a |
| **Klaus** | 25304.000 b | 9725.333 a | 14040.889 b | 89842.000 a | 19915.667 b | 41744.889 a | 23243.778 c | 21070.778 b |
| **Montagna** | 27685.750 b | 8210.000 b | 13821.625 b | 15944.375 b | 15798.250 c | 19460.875 c | 47447.000 b | 12159.875 c |
| **Pr > F(Model)** | <0.0001 | 0.033 | 0.017 | 0.001 | <0.0001 | <0.0001 | <0.0001 | <0.0001 |
| **Significant** | Yes | Yes | Yes | Yes | Yes | Yes | Yes | Yes |

**Table S12.** Oenological parameters of musts samples of Aldino vineyards. C: Control/Alcoholic fermentation; F: Frozen.

| **Vineyard** | Glucose-Fructose (g^.^L^-1^ ) | Tartaric acid (g^.^L^-1^ ) | Acetic acid (g^.^L^-1^ ) | Malic acid (g^.^L^-1^ ) | Lactic acid (g^.^L^-1^ ) | Alfa amino nitrogen (mg^.^L^-1^ ) | Ammonia nitrogen (mg^.^L^-1^ ) |
| --- | --- | --- | --- | --- | --- | --- | --- |
| **V_C** | 208.49 ± 0.01 | 2.90± 0.01 | 0.16± 0.01 | 3.88± 0.01 | **** | 132± 1 | 89± 1 |
| **V_F** | 248.67± 0.01 | 1.00± 0.01 | 0.18± 0.01 | 4.09± 0.01 | **** | 151± 1 | 85± 1 |

**** = values below the quantitation threshold

**Table S13.** Oenological parameters of young wines samples from Aldino vineyard. C:Control; F: Frozen; NF: Non-frozen; LL: Simultaneous alcoholic and malolactic fermentation.

| **Vineyard** | Glucose-Fructose (g^.^L^-1^ ) | Tartaric acid (g^.^L^-1^ ) | Acetic acid (g^.^L^-1^ ) | Malic acid (g^.^L^-1^ ) | Lactic acid (g^.^L^-1^ ) | Total SO_2_ (mg^.^L^-1^ ) | Free SO_2_ (mg^.^L^-1^ ) | Total polyphenols (mg^.^L^-1^ ) |
| --- | --- | --- | --- | --- | --- | --- | --- | --- |
| **V_NF_C** | **** | 2.69 ± 0.01 | 0.31 ± 0.01 | 3.71 ± 0.01 | **** | 60.00 ± 1 | 13.00 ± 1 | 445.05 ± 1 |
| **V_F_C** | 7.83 | 1.86 ± 0.01 | 0.39 ± 0.01 | 4.18 ± 0.01 | **** | 35.00 ± 1 | 1.00 ± 1 | 427.85 ± 1 |
| **V_NF_LL** | 0.37 | 2.89 ± 0.01 | 0.31 ± 0.01 | 0.16 ± 0.01 | 2.64 | 56 00± 1 | 7.00 ± 1 | 462.25 ± 1 |
| **V_F_LL** | 7.92 | 1.62 ± 0.01 | 0.39 ± 0.01 | 0.05 ± 0.01 | 2.98 | 25.00 ± 1 | 1.00 ± 1 | 430.00 ± 1 |

**** = values below the quantitation threshold

**Table S14**. One-way ANOVA of the volatile compounds present in the must samples of Aldino vineyard.

| **Category** | **II** | **III** | **V** | **VI** | **XIII** | **XVII** | **XXXVIII** | **XL** | **XLIV** |
| --- | --- | --- | --- | --- | --- | --- | --- | --- | --- |
| **Frozen** | 3290110.333 b | 4950867.000 b | 2225110.000 b | 24465721.667 b | 162941735.000 a | 60553196.000 a | 165694.667 a | 1405790.667 b | 3213572.000 a |
| **Control treatment** | 4935945.333 a | 17143279.000 a | 3076046.000 a | 60987701.000 a | 76644925.000 b | 44032298.333 b | 15138.667 b | 1906867.333 a | 1548570.667 b |
| **Pr > F(Model)** | 0.014 | 0.021 | 0.019 | 0.001 | 0.007 | 0.049 | 0.002 | 0.015 | 0.039 |
| **Significant** | Yes | Yes | Yes | Yes | Yes | Yes | Yes | Yes | Yes |

**Table S15**. Two-way ANOVA of the volatile compounds present in the young wines of Aldino vineyard.

| **Category** | **II** | **VII** | **VIII** | **IX** | **XI** | **XII** | **XIV** |
| --- | --- | --- | --- | --- | --- | --- | --- |
| **Non Frozen*Yeast and MLF bacteria** | 11891511.333 a | 329976074.667 a | 58678381.333 a | 773525591.333 b | 123141846.000 a | 7605901.000 a | 175295.000 c |
| **Frozen *Yeast and MLF bacteria** | 5605423.333 b | 129714826.333 b | 15575292.000 b | 1167078916.667 a | 40426091.333 b | 1910550.333 b | 199920.333 bc |
| **Non Frozen*Normal Yeast** | 6487969.333 b | 129915457.667 b | 17987852.333 b | 1264708921.333 a | 64452632.333 b | 4311586.000 b | 294787.667 ab |
| **Frozen *Normal Yeast** | 5416719.000 b | 115972452.333 b | 14172266.000 b | 1120292612.667 a | 36341627.000 b | 1825240.333 b | 318923.000 a |
| **Pr > F(Model)** | 0.019 | 0.030 | 0.040 | 0.028 | 0.024 | 0.005 | 0.043 |
| **Significant** | Yes | Yes | Yes | Yes | Yes | Yes | Yes |

| **Category** | **XVX** | **XXI** | **XXII** | **XXXV** | **XXXIX** | **XL** | **LII** |
| --- | --- | --- | --- | --- | --- | --- | --- |
| **Non Frozen*Yeast and MLF bacteria** | 4261159.000 b | 393753.667 a | 832137.333 b | 48827672.333 b | 237455262.667 a | 3397388.667 a | 556916.667 c |
| **Frozen *Yeast and MLF bacteria** | 6166133.000 a | 181797.667 b | 1460682.667 a | 101987094.667 a | 165202797.000 b | 2587361.000 b | 1542395.000 a |
| **Non Frozen*Normal Yeast** | 3860266.667 b | 324198.333 ab | 1410367.667 a | 56600675.667 b | 223846895.333 a | 3174290.333 a | 997826.667 b |
| **Frozen *Normal Yeast** | 6373458.000 a | 183524.000 b | 1200382.667 ab | 103468073.333 a | 168641585.333 b | 2502901.000 b | 1009107.667 b |
| **Pr > F(Model)** | 0.030 | 0.045 | 0.047 | 0.000 | 0.022 | 0.015 | 0.000 |
| **Significant** | Yes | Yes | Yes | Yes | Yes | Yes | Yes |

**Table S16**. One-way ANOVA of the phenolic compounds present in the must samples of Aldino vineyard.

| **Category** | **x29** | **x30** | **x33** | **x38** | **x39** | **x40** | **x45** | **x47** | **x51** |
| --- | --- | --- | --- | --- | --- | --- | --- | --- | --- |
| **Control treatment** | 138407.333 a | 35476.000 a | 782686.333 a | 69842.333 a | 20305.000 a | 95422.000 a | 29396.333 a | 37642.667 a | 24375.000 a |
| **Frozen** | 53625.000 b | 13347.000 b | 245837.667 b | 27639.333 b | 11444.000 b | 29918.000 b | 12266.000 b | 16629.667 b | 15909.333 b |
| **Pr > F(Model)** | 0.008 | 0.006 | 0.005 | 0.009 | 0.013 | 0.006 | 0.007 | 0.001 | 0.020 |
| **Significant** | Yes | Yes | Yes | Yes | Yes | Yes | Yes | Yes | Yes |

**Table S17**. Two-way ANOVA of the phenolic compounds present in the young wines of Aldino vineyard.

| **Category** | **x2** | **x4** | **x13** | **x17** | **x19** | **x25** | **x26** | **x27** | **x30** | **x31** |
| --- | --- | --- | --- | --- | --- | --- | --- | --- | --- | --- |
| **Non Frozen*Normal Yeast** | 8396.000 b | 13478.667 bc | 18053.333 ab | 149141.000 a | 60605.333 bc | 14011.333 b | 21033.667 ab | 45660.000 b | 34760.000 a | 7542.000 b |
| **Non Frozen*Yeast and MLF bacteria** | 11784.167 ab | 42694.833 a | 22279.500 ab | 148263.500 a | 50477.000 c | 16878.167 b | 22453.333 a | 49334.333 b | 29246.333 ab | 7930.167 b |
| **Frozen *Yeast and MLF bacteria** | 15171.000 a | 25457.667 b | 14020.000 b | 122266.833 b | 92326.500 a | 24687.000 a | 20174.333 b | 69857.000 a | 18157.833 c | 15237.000 a |
| **Frozen *Normal Yeast** | 8905.333 b | 11538.222 c | 30509.333 a | 140626.889 a | 74670.222 ab | 21075.889 ab | 21650.000 a | 61536.667 a | 23080.667 bc | 11118.222 ab |
| **Pr > F(Model)** | 0.021 | <0.0001 | 0.041 | 0.000 | 0.008 | 0.028 | 0.010 | 0.004 | 0.003 | 0.040 |
| **Significant** | Yes | Yes | Yes | Yes | Yes | Yes | Yes | Yes | Yes | Yes |

| **Category** | **x33** | **x36** | **x39** | **x42** | **x43** | **x45** | **x46** | **x47** | **x49** |
| --- | --- | --- | --- | --- | --- | --- | --- | --- | --- |
| **Non Frozen*Normal Yeast** | 725374.333 a | 107404.667 a | 13005.333 a | 19687.333 a | 27830.333 ab | 24038.333 a | 10809.000 a | 33373.333 a | 13114.000 a |
| **Non Frozen*Yeast and MLF bacteria** | 644174.833 a | 88295.167 c | 9919.667 b | 16976.833 ab | 44499.833 a | 21693.000 ab | 10452.667 a | 30991.167 ab | 15745.500 a |
| **Frozen *Yeast and MLF bacteria** | 345589.667 b | 96776.333 b | 9285.000 b | 13469.833 b | 15890.167 b | 13374.000 c | 8289.167 b | 26593.833 c | 5363.000 b |
| **Frozen *Normal Yeast** | 455766.667 b | 95468.000 b | 9285.000 b | 17235.778 a | 28327.222 ab | 18072.778 b | 9590.222 a | 28444.333 bc | 8297.000 b |
| **Pr > F(Model)** | <0.0001 | 0.004 | <0.0001 | 0.045 | 0.016 | 0.001 | 0.009 | 0.005 | <0.0001 |
| **Significant** | Yes | Yes | Yes | Yes | Yes | Yes | Yes | Yes | Yes |

**Table S18.** One-way ANOVA of the sensory attributes in the bottled wines of different vineyards at storage time 0.

| **Category** | **clarity** | **colour-tonality** | **colour intensity** | **overall-intensity** | **olfactory-citrus-fruit** | **olfactory-pome-tree-fruit** | **olfactory-yellow-tree-fruit** | **olfactory-tropical-fruit** | **olfactory-fresh-vegetative** | **olfactory-nutty** | **olfactory-floral** | **olfactory-spicy** | **olfactory-cleanness/unpleasant odours** |
| --- | --- | --- | --- | --- | --- | --- | --- | --- | --- | --- | --- | --- | --- |
| **Montagna** | 5.650 b | 3.000 a | 3.000 a | 6.450 a | 3.250 b | 3.800 a | 3.550 a | 2.950 a | 2.050 a | 2.450 a | 2.800 a | 2.450 a | 7.550 b |
| **Aldino** | 8.167 a | 2.778 ab | 3.278 a | 7.222 a | 3.000 b | 2.722 a | 3.222 ab | 2.833 a | 2.389 a | 2.000 ab | 3.222 a | 2.333 a | 8.056 a |
| **Klaus** | 2.056 c | 2.222 b | 2.667 a | 6.278 a | 4.056 a | 2.889 a | 2.444 b | 3.111 a | 1.500 a | 1.833 b | 2.278 a | 1.833 a | 6.611 c |
| **Pr > F(Model)** | 0.000 | 0.069 | 0.204 | 0.217 | 0.036 | 0.102 | 0.058 | 0.289 | 0.310 | 0.072 | 0.284 | 0.113 | 0.004 |
| **Significant** | Yes | No | No | No | Yes | No | No | No | No | No | No | No | Yes |

| **Category** | **acidity-sourness** | **saltiness-sapidity** | **bitterness** | **sweetness** | **astringency-tannicity** | **warmness** | **gustatory-citrus fruit** | **gustatory-pome tree fruit** | **gustatory-yellow tree fruit** | **gustatory-spicy** | **gustatory-cleanness/unpleasant odours** | **overall-quality** |
| --- | --- | --- | --- | --- | --- | --- | --- | --- | --- | --- | --- | --- |
| **Montagna** | 5.600 a | 4.400 a | 3.550 a | 2.050 a | 2.350 a | 4.600 a | 4.000 a | 2.750 a | 2.550 a | 2.750 a | 6.900 ab | 6.200 b |
| **Aldino** | 6.333 a | 4.444 a | 2.667 a | 2.222 a | 2.111 a | 4.222 a | 4.444 a | 2.167 b | 2.389 a | 2.556 a | 7.833 a | 7.444 a |
| **Klaus** | 6.056 a | 4.000 a | 3.667 a | 2.222 a | 2.278 a | 4.222 a | 3.556 a | 2.389 ab | 2.389 a | 2.000 b | 5.944 b | 4.889 c |
| **Pr > F(Model)** | 0.275 | 0.450 | 0.288 | 0.890 | 0.893 | 0.373 | 0.400 | 0.065 | 0.534 | 0.010 | 0.107 | 0.015 |
| **Significant** | No | No | No | No | No | No | No | No | No | Yes | No | Yes |

**Table S19.** One-way ANOVA of the sensory attributes in the bottled wines of different vineyards at storage time 6.

| **Category** | **clarity** | **colour-tonality** | **colour intensity** | **overall-intensity** | **olfactory-citrus-fruit** | **olfactory-pome-tree-fruit** | **olfactory-yellow-tree-fruit** | **olfactory-tropical-fruit** | **olfactory-fresh-vegetative** | **olfactory-nutty** | **olfactory-floral** | **olfactory-spicy** | **olfactory-cleanness/unpleasant odours** |
| --- | --- | --- | --- | --- | --- | --- | --- | --- | --- | --- | --- | --- | --- |
| **Montagna** | 8.318 a | 3.045 a | 2.545 a | 6.045 a | 2.364 a | 3.545 a | 3.773 a | 3.000 a | 2.091 a | 2.636 a | 2.864 a | 2.182 a | 8.000 a |
| **Aldino** | 8.400 a | 3.650 a | 2.800 a | 6.750 a | 1.950 a | 3.650 a | 3.700 a | 3.150 a | 2.000 a | 2.400 a | 2.650 ab | 2.300 a | 6.900 b |
| **Klaus** | 8.250 a | 2.500 a | 1.900 a | 6.450 a | 2.400 a | 3.250 a | 3.100 a | 2.750 a | 2.450 a | 2.600 a | 1.950 b | 1.750 a | 7.100 b |
| **Pr > F(Model)** | 0.655 | 0.128 | 0.183 | 0.464 | 0.313 | 0.786 | 0.352 | 0.156 | 0.405 | 0.652 | 0.064 | 0.301 | 0.039 |
| **Significant** | No | No | No | No | No | No | No | No | No | No | No | No | Yes |

| **Category** | **acidity-sourness** | **saltiness-sapidity** | **bitterness** | **sweetness** | **astringency-tannicity** | **warmness** | **gustatory-citrus fruit** | **gustatory-pome tree fruit** | **gustatory-yellow tree fruit** | **gustatory-spicy** | **gustatory-cleanness/unpleasant odours** | **overall-quality** |
| --- | --- | --- | --- | --- | --- | --- | --- | --- | --- | --- | --- | --- |
| **Montagna** | 4.773 a | 3.227 a | 2.773 a | 2.000 a | 2.682 a | 4.364 a | 3.227 ab | 3.227 a | 2.909 a | 2.545 a | 7.500 a | 6.864 a |
| **Aldino** | 4.500 a | 3.700 a | 2.550 a | 2.150 a | 2.350 a | 4.250 a | 3.850 a | 2.850 b | 2.550 ab | 2.400 a | 6.450 b | 6.650 ab |
| **Klaus** | 4.450 a | 3.250 a | 3.400 a | 2.150 a | 2.300 a | 4.500 a | 2.900 b | 2.700 b | 2.050 b | 2.200 a | 6.600 b | 6.200 b |
| **Pr > F(Model)** | 0.357 | 0.392 | 0.169 | 0.919 | 0.453 | 0.456 | 0.078 | 0.027 | 0.028 | 0.582 | 0.059 | 0.066 |
| **Significant** | No | No | No | No | No | No | No | Yes | Yes | No | No | No |

**Table S20.** One-way ANOVA of the sensory attributes in the bottled wines of different vineyards at storage time 12.

| **Category** | **clarity** | **colour-tonality** | **colour intensity** | **overall-intensity** | **olfactory-citrus-fruit** | **olfactory-pome-tree-fruit** | **olfactory-yellow-tree-fruit** | **olfactory-tropical-fruit** | **olfactory-fresh-vegetative** | **olfactory-nutty** | **olfactory-floral** | **olfactory-spicy** | **olfactory-cleanness/unpleasant odours** |
| --- | --- | --- | --- | --- | --- | --- | --- | --- | --- | --- | --- | --- | --- |
| **Aldino** | 8.500 a | 4.750 a | 4.500 a | 6.625 a | 2.625 a | 3.875 a | 3.625 a | 2.375 b | 2.000 ab | 3.500 a | 3.875 a | 4.125 a | 8.250 a |
| **Klaus** | 8.375 a | 4.000 ab | 2.250 a | 6.250 a | 2.125 b | 4.125 a | 3.875 a | 2.375 b | 2.250 a | 2.875 b | 2.625 a | 2.250 b | 6.250 b |
| **Montagna** | 8.357 a | 3.500 b | 3.357 a | 6.429 a | 2.429 ab | 4.000 a | 3.429 a | 3.429 a | 1.500 b | 2.071 c | 3.143 a | 1.929 b | 7.857 a |
| **Pr > F(Model)** | 0.501 | 0.053 | 0.110 | 0.870 | 0.088 | 0.816 | 0.386 | 0.017 | 0.082 | 0.003 | 0.394 | 0.011 | 0.007 |
| **Significant** | No | No | No | No | No | No | No | Yes | No | Yes | No | Yes | Yes |

| **Category** | **acidity-sourness** | **saltiness-sapidity** | **bitterness** | **sweetness** | **astringency-tannicity** | **warmness** | **gustatory-citrus fruit** | **gustatory-pome tree fruit** | **gustatory-yellow tree fruit** | **gustatory-spicy** | **gustatory-cleanness/unpleasant odours** | **overall-quality** |
| --- | --- | --- | --- | --- | --- | --- | --- | --- | --- | --- | --- | --- |
| **Aldino** | 5.000 a | 2.625 a | 1.875 a | 2.125 b | 2.250 a | 4.250 a | 3.250 a | 3.750 a | 3.500 a | 4.500 a | 8.250 a | 7.625 a |
| **Klaus** | 4.625 ab | 2.125 a | 1.625 a | 2.500 a | 1.750 a | 4.875 a | 3.375 a | 3.875 a | 3.125 a | 4.250 a | 7.750 b | 6.375 b |
| **Montagna** | 4.214 b | 3.143 a | 1.929 a | 2.500 a | 2.000 a | 4.286 a | 3.214 a | 3.786 a | 2.429 a | 2.500 b | 8.000 ab | 7.571 a |
| **Pr > F(Model)** | 0.098 | 0.128 | 0.262 | 0.077 | 0.192 | 0.494 | 0.939 | 0.867 | 0.143 | 0.012 | 0.053 | 0.005 |
| **Significant** | No | No | No | No | No | No | No | No | No | Yes | No | Yes |

**Table S21.** Two-way ANOVA of the sensory attributes in the bottled wines of Aldino vineyard at storage time 0.

| **Category** | **clarity** | **colour-tonality** | **colour intensity** | **overall-intensity** | **olfactory-citrus-fruit** | **olfactory-pome-tree-fruit** | **olfactory-yellow-tree-fruit** | **olfactory-tropical-fruit** | **olfactory-fresh-vegetative** | **olfactory-nutty** | **olfactory-floral** | **olfactory-spicy** | **olfactory-cleanness/unpleasant odours** |
| --- | --- | --- | --- | --- | --- | --- | --- | --- | --- | --- | --- | --- | --- |
| **Non Frozen*Yeast and MLF bacteria** | 8.300 a | 3.500 a | 3.600 a | 6.850 ab | 3.450 a | 3.300 ab | 2.850 a | 3.450 a | 2.450 a | 2.150 a | 3.250 a | 2.100 a | 7.100 b |
| **Frozen *Normal Yeast** | 6.700 b | 3.200 a | 3.550 a | 6.150 b | 3.750 a | 3.800 a | 3.150 a | 3.200 a | 2.150 a | 2.200 a | 2.800 a | 2.150 a | 7.700 ab |
| **Frozen *Yeast and MLF bacteria** | 8.278 a | 3.389 a | 3.556 a | 7.056 ab | 3.222 a | 3.278 ab | 3.389 a | 3.389 a | 2.056 a | 2.389 a | 3.000 a | 1.833 a | 7.944 ab |
| **Non Frozen*Normal Yeast** | 8.167 a | 2.778 b | 3.278 a | 7.222 a | 3.000 a | 2.722 b | 3.222 a | 2.833 a | 2.389 a | 2.000 a | 3.222 a | 2.333 a | 8.056 a |
| **Pr > F(Model)** | 0.006 | 0.020 | 0.731 | 0.115 | 0.230 | 0.048 | 0.487 | 0.361 | 0.809 | 0.314 | 0.487 | 0.550 | 0.117 |
| **Significant** | Yes | Yes | No | No | No | Yes | No | No | No | No | No | No | No |

| **Category** | **acidity-sourness** | **saltiness-sapidity** | **bitterness** | **sweetness** | **astringency-tannicity** | **warmness** | **gustatory-citrus fruit** | **gustatory-pome tree fruit** | **gustatory-yellow tree fruit** | **gustatory-spicy** | **gustatory-cleanness/unpleasant odours** | **overall-quality** |
| --- | --- | --- | --- | --- | --- | --- | --- | --- | --- | --- | --- | --- |
| **Non Frozen*Yeast and MLF bacteria** | 6.050 ab | 4.250 a | 3.450 a | 2.350 a | 2.350 a | 5.400 a | 4.400 a | 2.850 b | 2.350 a | 2.350 a | 6.950 a | 6.500 b |
| **Frozen *Normal Yeast** | 5.800 ab | 4.250 a | 3.400 a | 2.650 a | 2.400 a | 5.000 a | 4.500 a | 3.250 a | 2.600 a | 2.350 a | 6.950 a | 6.550 b |
| **Frozen *Yeast and MLF bacteria** | 5.444 b | 3.889 a | 3.389 a | 2.611 a | 2.333 a | 4.667 a | 3.389 a | 2.889 ab | 2.667 a | 2.167 a | 7.389 a | 7.278 a |
| **Non Frozen*Normal Yeast** | 6.333 a | 4.444 a | 2.667 a | 2.222 a | 2.111 a | 4.222 a | 4.444 a | 2.167 c | 2.389 a | 2.556 a | 7.833 a | 7.444 a |
| **Pr > F(Model)** | 0.159 | 0.628 | 0.237 | 0.724 | 0.744 | 0.188 | 0.160 | 0.007 | 0.762 | 0.450 | 0.139 | 0.037 |
| **Significant** | No | No | No | No | No | No | No | Yes | No | No | No | Yes |

**Table S22.** Two-way ANOVA of the sensory attributes in the bottled wines of Aldino vineyard at storage time 6.

| **Category** | **clarity** | **colour-tonality** | **colour intensity** | **overall-intensity** | **olfactory-citrus-fruit** | **olfactory-pome-tree-fruit** | **olfactory-yellow-tree-fruit** | **olfactory-tropical-fruit** | **olfactory-fresh-vegetative** | **olfactory-nutty** | **olfactory-floral** | **olfactory-spicy** | **olfactory-cleanness/unpleasant odours** |
| --- | --- | --- | --- | --- | --- | --- | --- | --- | --- | --- | --- | --- | --- |
| **Non Frozen*Yeast and MLF bacteria** | 8.273 a | 3.818 a | 3.500 a | 6.318 a | 2.455 a | 3.409 a | 3.273 a | 3.227 a | 2.864 a | 3.000 a | 3.091 a | 2.409 a | 7.773 ab |
| **Frozen *Normal Yeast** | 8.045 a | 4.182 a | 3.636 a | 6.091 a | 2.409 a | 3.364 a | 3.682 a | 3.409 a | 1.955 a | 2.409 b | 2.909 a | 2.045 a | 7.864 a |
| **Frozen *Yeast and MLF bacteria** | 7.950 a | 3.850 a | 2.900 a | 6.900 a | 2.150 a | 3.850 a | 3.400 a | 3.350 a | 2.500 a | 2.600 ab | 2.450 a | 2.900 a | 6.950 ab |
| **Non Frozen*Normal Yeast** | 8.400 a | 3.650 a | 2.800 a | 6.750 a | 1.950 a | 3.650 a | 3.700 a | 3.150 a | 2.000 a | 2.400 b | 2.650 a | 2.300 a | 6.900 b |
| **Pr > F(Model)** | 0.302 | 0.607 | 0.204 | 0.229 | 0.390 | 0.741 | 0.384 | 0.744 | 0.198 | 0.084 | 0.493 | 0.215 | 0.083 |
| **Significant** | No | No | No | No | No | No | No | No | No | No | No | No | No |

| **Category** | **acidity-sourness** | **saltiness-sapidity** | **bitterness** | **sweetness** | **astringency-tannicity** | **warmness** | **gustatory-citrus fruit** | **gustatory-pome tree fruit** | **gustatory-yellow tree fruit** | **gustatory-spicy** | **gustatory-cleanness/unpleasant odours** | **overall-quality** |
| --- | --- | --- | --- | --- | --- | --- | --- | --- | --- | --- | --- | --- |
| **Non Frozen*Yeast and MLF bacteria** | 4.773 a | 3.545 a | 2.727 a | 2.409 a | 2.727 a | 4.045 a | 3.591 ab | 3.318 a | 2.727 ab | 2.545 a | 7.818 a | 7.182 a |
| **Frozen *Normal Yeast** | 4.773 a | 3.545 a | 2.727 a | 2.545 a | 2.727 a | 4.273 a | 3.500 b | 3.227 ab | 3.318 a | 2.545 a | 7.545 ab | 7.136 a |
| **Frozen *Yeast and MLF bacteria** | 4.050 a | 2.900 b | 3.050 a | 2.450 a | 2.500 a | 4.050 a | 2.750 c | 3.250 ab | 2.700 ab | 2.500 a | 6.800 ab | 6.350 b |
| **Non Frozen*Normal Yeast** | 4.500 a | 3.700 a | 2.550 a | 2.150 a | 2.350 a | 4.250 a | 3.850 a | 2.850 b | 2.550 b | 2.400 a | 6.450 b | 6.650 ab |
| **Pr > F(Model)** | 0.395 | 0.084 | 0.620 | 0.808 | 0.591 | 0.590 | 0.003 | 0.111 | 0.150 | 0.922 | 0.124 | 0.098 |
| **Significant** | No | No | No | No | No | No | Yes | No | No | No | No | No |

**Table S23.** Two-way ANOVA of the sensory attributes in the bottled wines of Aldino vineyard at storage time 12.

| **Category** | **clarity** | **colour-tonality** | **colour intensity** | **overall-intensity** | **olfactory-citrus-fruit** | **olfactory-pome-tree-fruit** | **olfactory-yellow-tree-fruit** | **olfactory-tropical-fruit** | **olfactory-fresh-vegetative** | **olfactory-nutty** | **olfactory-floral** | **olfactory-spicy** | **olfactory-cleanness/unpleasant odours** |
| --- | --- | --- | --- | --- | --- | --- | --- | --- | --- | --- | --- | --- | --- |
| **Non Frozen*Yeast and MLF bacteria** | 8.500 a | 4.750 a | 4.500 a | 6.625 a | 2.625 a | 3.875 a | 3.625 a | 2.375 c | 2.000 ab | 3.500 a | 3.875 a | 4.125 a | 8.250 a |
| **Frozen *Normal Yeast** | 8.643 a | 4.500 a | 3.786 a | 6.929 a | 2.071 a | 4.071 a | 3.214 a | 3.429 a | 1.500 b | 2.571 a | 3.143 a | 2.143 b | 7.714 a |
| **Frozen *Yeast and MLF bacteria** | 8.357 a | 4.357 a | 3.571 a | 7.286 a | 2.143 a | 4.357 a | 2.929 a | 3.071 ab | 2.143 a | 2.643 a | 2.857 a | 2.571 ab | 7.357 a |
| **Non Frozen*Normal Yeast** | 8.375 a | 4.875 a | 3.875 a | 6.625 a | 2.000 a | 3.875 a | 3.500 a | 2.625 bc | 1.750 ab | 2.750 a | 3.000 a | 3.750 a | 7.375 a |
| **Pr > F(Model)** | 0.180 | 0.302 | 0.608 | 0.618 | 0.360 | 0.403 | 0.769 | 0.030 | 0.083 | 0.179 | 0.336 | 0.068 | 0.658 |
| **Significant** | No | No | No | No | No | No | No | Yes | No | No | No | No | No |

| **Category** | **acidity-sourness** | **saltiness-sapidity** | **bitterness** | **sweetness** | **astringency-tannicity** | **warmness** | **gustatory-citrus fruit** | **gustatory-pome tree fruit** | **gustatory-yellow tree fruit** | **gustatory-spicy** | **gustatory-cleanness/unpleasant odours** | **overall-quality** |
| --- | --- | --- | --- | --- | --- | --- | --- | --- | --- | --- | --- | --- |
| **Non Frozen*Yeast and MLF bacteria** | 5.000 a | 2.625 ab | 1.875 a | 2.125 a | 2.250 a | 4.250 a | 3.250 a | 3.750 a | 3.500 a | 4.500 a | 8.250 b | 7.625 a |
| **Frozen *Normal Yeast** | 5.000 a | 3.286 a | 2.143 a | 2.643 a | 2.000 a | 4.429 a | 3.429 a | 4.286 a | 3.571 a | 2.714 c | 7.786 c | 7.643 a |
| **Frozen *Yeast and MLF bacteria** | 4.214 a | 3.214 a | 2.286 a | 2.714 a | 2.071 a | 4.643 a | 3.357 a | 3.929 a | 3.286 a | 3.000 bc | 7.714 c | 7.357 a |
| **Non Frozen*Normal Yeast** | 5.000 a | 2.250 b | 2.000 a | 2.625 a | 1.625 a | 4.625 a | 2.625 a | 3.625 a | 3.500 a | 3.750 ab | 8.500 a | 7.500 a |
| **Pr > F(Model)** | 0.333 | 0.096 | 0.226 | 0.609 | 0.382 | 0.840 | 0.462 | 0.221 | 0.929 | 0.011 | 0.000 | 0.908 |
| **Significant** | No | No | No | No | No | No | No | No | No | Yes | Yes | No |

**Figure S14**. The variables importance in projection (VIP) – (A) and effects of the variables (B) in the PLS regression olfactory attribute “floral” aroma vs volatile compounds for Pinot Blanc

**Figure S15**. The variables importance in projection (VIP) in the PLS regression visual and gustatory attribute “clarity”, “colour tonality”, “colour intensity” and “bitterness” vs phenolic compounds for Pinot Blanc**.**

| **** |
| --- |

**Figure S16**. The effects of the variables on the PLS equation of visual and gustatory attribute “clarity”, “colour tonality”, “colour intensity” and “bitterness” for Pinot Blanc**.**

**Table S24.** Quality index for the PLS regression on visual and gustatory sensory variables for Pinot Blanc wines

|  | **clarity** | **colour-tonality** | **colour-intensity** | **bitterness** |
| --- | --- | --- | --- | --- |
| **R^2^** | 0.846 | 0.772 | 0.676 | 0.586 |
| **Standard deviation** | 0.647 | 0.384 | 0.418 | 0.463 |
| **MSE** | 0.372 | 0.131 | 0.156 | 0.190 |
| **RMSE** | 0.610 | 0.362 | 0.395 | 0.436 |
